# Phenotypic and Immunological Characterization of Patients with Activated PI3Kδ Syndrome 1 Presenting with Autoimmunity

**DOI:** 10.1007/s10875-024-01705-w

**Published:** 2024-04-18

**Authors:** Qifan Li, Wenjie Wang, Qi Wu, Qinhua Zhou, Wenjing Ying, Xiaoying Hui, Bijun Sun, Jia Hou, Feng Qian, Xiaochuan Wang, Jinqiao Sun

**Affiliations:** 1 Department of Clinical Immunology, National Children Medical Center, Children’s Hospital of Fudan University, Shanghai, 201102 China; 2 Shanghai Institute of Infectious Disease and Biosecurity, Shanghai, 200032 China; 3 Ministry of Education Key Laboratory of Contemporary Anthropology, Human Phenome Institute, School of Life Sciences, Fudan University, Shanghai, 200438 China

**Keywords:** Activated PI3Kδ Syndrome 1, *PIK3CD*, Immune Phenotypes, Autoantigen Microarray

## Abstract

**Purpose:**

Autoimmunity is a significant feature of APDS1 patients. We aimed to explore the pathogenic immune phenotype and possible mechanisms of autoimmunity in APDS1 patients.

**Methods:**

The clinical records and laboratory data of 42 APDS1 patients were reviewed. Immunophenotypes were evaluated by multiparametric flow cytometry. Autoantibodies were detected via antigen microarray analysis.

**Results:**

A total of 42 children with *PIK3CD* gene mutations were enrolled. Immunological tests revealed increased proportions of effector memory cells (86%) and central memory cells (59%) among CD4+ T cells; increased proportions of effector memory cells (83%) and terminally differentiated effector memory T cells (38%) among CD8+ T cells. Fewer CD3+ T cells and B cells and higher IgG levels were reported in patients with autoimmunity. The proportion of Tregs was decreased, and the proportions of Th9, Tfh, and Tfr cells were increased in APDS1 patients. Among APDS1 patients, higher proportion of Th2 and Tfr cells were found in those with autoimmunity. The proportions of CD11c+ B and CD21lo B cells in patients with autoimmunity were significantly increased. Antigen microarray analysis revealed a wide range of IgG/IgM autoantibodies in patients with APDS1. In patients with autoimmunity, the proportion of Tfr might be positively correlated with autoantibodies.

**Conclusions:**

The pathogenic immune phenotype of APDS1 patients included (1) deceased CD3+ T-cell and B-cell counts and increased IgG levels in patients with autoimmunity, (2) an imbalanced T helper cell subset, (3) increased proportions of autoreactive B cells, and (4) distinct autoantibody reactivities in patients with autoimmunity.

**Supplementary Information:**

The online version contains supplementary material available at 10.1007/s10875-024-01705-w.

## Introduction

The PI3K/AKT/mTOR signaling pathway is a highly conserved signal transduction network in immune cells that promotes cell survival, growth, and proliferation [1, 2]. PI3Kδ is a heterodimer composed of the catalytic subunit p110δ (encoded by *PIK3CD*) that constitutively associates with the regulatory subunit p85α (encoded by *PIK3R1*) [3, 4]. Heterozygous gain-of-function mutations in *PIK3CD* and loss-of-function *PIK3R1* cause predominantly antibody deficiencies and are referred to as activated PI3Kδ syndrome 1(APDS1) and activated PI3Kδ syndrome 2(APDS2), respectively [5–8].

The latest classification of human inborn errors of immunity (IEI) assigns APDS1 into a group with a common variable immunodeficiency (CVID) phenotype [9]. Although patients with APDS1 present with different clinical manifestations and symptoms, APDS1 is characterized by recurrent respiratory tract infections, lymphadenopathy, hepatosplenomegaly, persistent Epstein–Barr virus (EBV)/cytomegalovirus viraemia (CMV), hyper-IgM syndrome, and increased numbers of senescent T cells [10]. Autoimmunity is frequently observed in patients with various IEI. Studies to date imply increased activity of PI3Kδ in multiple autoimmune diseases, such as rheumatoid arthritis [11]. Indeed, approximately 30% of APDS1 patients exhibit a variety of autoimmune conditions, including cytopenia (hemolytic anemia, neutropenia, and/or thrombocytopenia), inflammatory bowel disease (IBD), lupus-like nephritis, and rheumatologic diseases [12]. Previous studies revealed that B-cell dysfunction causes the expansion of self-reactive B cells and the production of autoreactive antibodies [13, 14].

An autoantigen microarray is a specific high-throughput proteome microarray for detecting a wide spectrum of autoantibodies. It is a valuable and powerful method for evaluating autoimmune phenomena and immune dysfunction. Previous studies have revealed that autoantibodies can exist prior to the onset of autoimmune diseases [15]. Early autoantibody screening may aid in the early diagnosis of APDS1 in patients with or without autoimmune disease.

In the present study, we investigated the immunological activity and autoimmunity of APDS1 patients. We obtained the clinical data of 42 APDS1 patients and applied multiparametric flow cytometry and antigen microarray analysis to identify the pathogenic lymphocyte phenotypes and autoantibodies in these patients. New perspectives are proposed in this paper to broaden the understanding of APDS1.

## Methods

The study was approved by the Ethics Committee of the Children’s Hospital of Fudan University (No. 2022 179). All patients’ guardians provided written informed consent for enrollment in this study.

### Patients and Clinical Data

Patients diagnosed with APDS1 on the basis of the presence of *PIK3CD* gene mutations confirmed by whole-exome sequencing, clinical manifestations and functional test findings were enrolled in our study. We obtained the clinical data of the patients. The methods for genetic testing and immunological function evaluation were the same as those described in our previous study [16].

### Multiparametric Flow Cytometry

High-throughput flow cytometry analyses were performed as described previously [17]. Briefly, 51 surface antibodies were divided into 6 panels. Peripheral whole blood was collected in ethylenediaminetetraacetic acid (EDTA) anticoagulant tubes. Whole-blood samples were cultured with antibodies at room temperature for 15 min, lysed with Lysing Solution (BD, USA), and washed with phosphate-buffered saline (PBS). After the final wash, the cells were resuspended in PBS. All samples were evaluated on a CytoFLEX LX Flow Cytometer (Beckman Coulter, USA) with CytExpert v2.4 (Beckman Coulter, USA).

### Antigen Microarray Analysis

Plasma samples from 15 patients and 12 controls were analyzed using an antigen microarray (GeneCopoeia, USA). The 120 IgG- and IgM-specific self-antigens that were analyzed are listed in Supplemental Table S7. Briefly, serum samples were first heat-inactivated and pretreated with DNAse I. Then, the samples were diluted 1:100 in PBS and applied to the arrays on the slide for hybridization with the antigens. The binding of IgG and IgM antibodies to antigens in the array was detected with Cy3-conjugated anti-human IgG (1/1000) and Cy5-conjugated anti-human IgM (1/1000) secondary antibodies. The slides were scanned with a LuxScan 10 K-A (CapitalBio Corporation, China) with laser wavelengths of 532 (for IgG) and 635 (for IgM) nm. Images in the array were converted to raw data with LuxScan_v3.0 (CapitalBio Corporation, China). The net fluorescent intensity (NFI) of each antigen was generated by subtracting the local background and negative control (PBS) signals. The signal/noise ratio (SNR = [foreground median - background median]/SD [background]) was calculated for each antigen. The NFI was normalized by a robust linear model using positive controls with different dilutions.

### Statistical Analysis

Flow cytometry data were analyzed with FlowJo (Treestar, Woodburn, OR). The measurement data are presented as the means, medians, ranges, and interquartile ranges (IQRs). For single comparisons of independent groups, the Mann‒Whitney test was performed, and a p value less than 0.05 was considered to indicate statistical significance. For comparisons of three independent groups, a Kruskal‒Wallis test was performed, and the Dunn test was used as a post hoc test. Statistical analysis was performed using GraphPad Prism (GraphPad Software, San Diego, CA, USA). Heatmaps were drawn with Heatmapper (www.heatmapper.ca) [18].

## Results

### Patient Characteristics

**Fig. 1:**
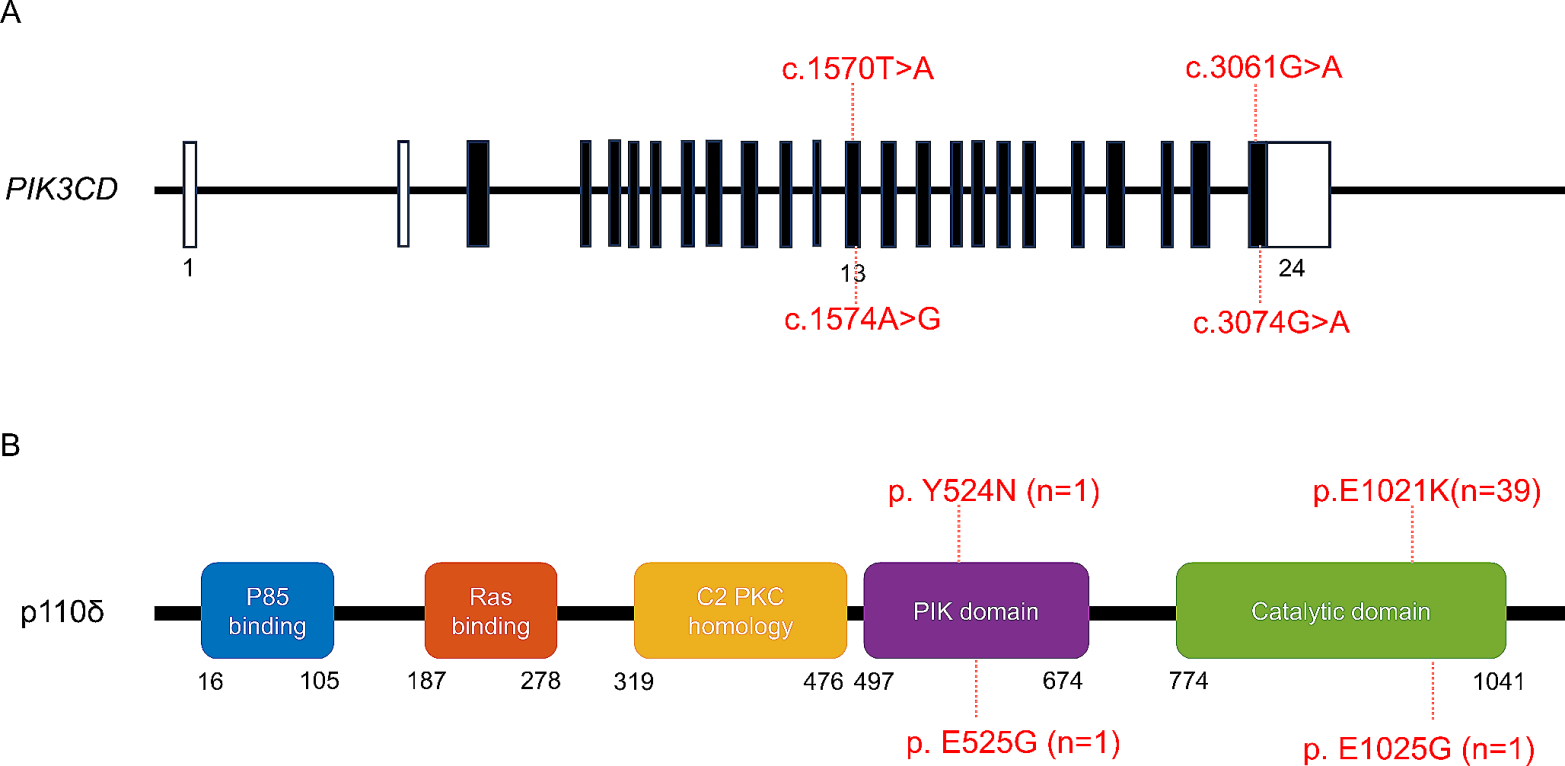
Mutations in *PIK3CD* and p110δ in 42 patients. **(A)** Sequence showing four kinds of mutations identified in the *PIK3CD* gene. Exons are in black, and introns are shown as thin lines. Noncoding regions are in white. **(B)** Location of mutations shown on the structural of p110δ. The mutations and affected patients in our study are in red

In the present study, we identified 42 Chinese APDS1 patients (27 males and 15 females). The clinical features of the 42 patients are shown in Table 1. The median age of onset of APDS1 was 14 months (range, 0.5 months to 8.5 years). The median age at genetic diagnosis was 66 months (range, 4 months to 15 years), and the diagnostic delay was 52 months. Among the 42 patients from 41 different families in China (P3 and P11 were from one family), 39 carried the previously described hotspot heterogeneous mutation c.3061G > A (p.E1021K) of the *PIK3CD* gene. Other mutations, including c.3074 A > G (p.E1025G), c.1570T > A (p.Y524N) and c.1574 A > G (p.E525G), were detected in three patients (Fig. 1). The patients in our cohort presented with a broad spectrum of clinical symptoms. Respiratory tract infections were the most common cause of hospital admission. Thirty-seven (88%) patients experienced recurrent respiratory infections. The median age at upper respiratory tract infection was 1.5 years (range, 0.5 months to 9 years). Twenty-two (59%) patients developed bronchiectasis after recurrent respiratory tract infections, and bronchiectasis typically developed 5.5 years (range, 3 months to 16 years) after infection. All patients experienced lymphadenomegaly, especially in the cervical, mediastinal, intrathoracic, coeliac, and inguinal lymph nodes. Thirty-nine patients (93%) had persistent or recurrent splenomegaly, and 30 patients (71%) had hepatomegaly. Seventeen patients (40%) had significant gastrointestinal symptoms; 3 patients (P19, P24 and P27) did not experience gastrointestinal symptoms, but colonoscopy revealed signs of IBD. Among our patients, 23 (55%) were considered to be affected by autoimmunity. Six patients had multiorgan tissue involvement. A diagnosis of IBD was considered in 12 patients. Autoimmunity involved the blood system of 10 patients, manifesting as hemolytic anemia and immune thrombocytopenia. Two patients were diagnosed with systemic lupus erythematosus (SLE), which resulted in kidney damage that progressed to lupus nephritis. Two patients were diagnosed with hypothyroidism. One patient was diagnosed with cutaneous vasculitis. One patient had joint pain and was diagnosed with arthritis after infection and other causes were ruled out; this patient was treated with adalimumab, which was effective.

**Table 1:** Characteristics of 42 APDS1 patients

ID	Sex	Age at Onset(Month)	Age at Onset(Month)	Mutation	Initial symptom	Sinopulmonaryinfections	Digestive symptoms	Lymphoproliferative	Autoimmunity	Other presentations	Therapy	Outcomes
P1	M	24	174	E1021K	Upper respiratory infection	Recurrent respiratory tract infections(2y)	Chronic diarrhea	Lymphadenomegaly		Atrial septal defect, lumbar disc herniation, nasosinusitis	anti-infection prophylaxis, IVIG, glucocorticoid, mTOR inhibitor	Alive
P2	F	1	161	E1021K	Upper respiratory infection	Recurrent respiratory tract infections(1m), PAH, bronchiectasis (16y)		Lymphadenomegaly, splenomegaly, hepatomegaly	Thrombocytopenia	Pericardial effusion, kidney injury, hypoalbuminemia, failure to thrive	anti-infection prophylaxis, IVIG, glucocorticoid, mTOR inhibitor	Died
P3	M	6	48	E1021K	Pneumonia	Recurrent respiratory tract infections(6m), bronchiectasis(7y)		Lymphadenomegaly, splenomegaly, hepatomegaly		Pericardial effusion, warts, proteinuria, hypoalbuminemia, intracranial hypertension, convulsion, failure to thrive, nasosinusitis, mastoiditis, brain atrophy	anti-infection prophylaxis, IVIG, glucocorticoid, mTOR inhibitor	Died
P4	M	106	164	E1021K	Splenomegaly	Recurrent respiratory tract infections(9y)		Lymphadenomegaly, splenomegaly	SLE, lupus nephritis, AIHA	Flat warts, urinary tract infection, failure to thrive	anti-infection prophylaxis, IVIG, glucocorticoid, mTOR inhibitor	Alive
P5	M	2	47	E1021K	Pneumonia	Recurrent respiratory tract infections(2m), otitis media, bronchiectasis(6y)	Gastroenteritis	Lymphadenomegaly, splenomegaly, hepatomegaly		Oblique inguinal hernia, urinary tract infection	anti-infection prophylaxis, IVIG, glucocorticoid, mTOR inhibitor, HSCT	Alive
P6	F	1	57	E1021K	Skin pigmentation	Recurrent respiratory tract infections(1y), otitis media		Lymphadenomegaly, splenomegaly, hepatomegaly		Failure to thrive	anti-infection prophylaxis, IVIG, mTOR inhibitor	Alive
P7	F	48	89	E1021K	Upper respiratory infection	Recurrent respiratory tract infections(4y), bronchiectasis, otitis media, eardrum perforation, hearing loss	Gastroenteritis	Lymphadenomegaly, splenomegaly, hepatomegaly	ITP	Conjunctivitis, failure to thrive	anti-infection prophylaxis, IVIG, mTOR inhibitor	Alive
P8	M	2	4	E1021K	Diarrhea	No	Chronic diarrhea	Lymphadenomegaly, splenomegaly			anti-infection prophylaxis, IVIG	Alive
P9	M	8	63	E1021K	Diarrhea	Recurrent respiratory tract infections(1.5y), otitis media, bronchiectasis(5y), pleural effusion	Chronic diarrhea	Lymphadenomegaly, splenomegaly, hepatomegaly		Congenital patent foramen ovale, hypoproteinemia, failure to thrive, nasosinusitis, mastoiditis, thick corpus callosum	anti-infection prophylaxis, IVIG, mTOR inhibitor	Alive
P10	M	1	104	E1025G	Purpura	Recurrent respiratory tract infections, bronchiectasis(9y)	Chronic diarrhea, colitis, ileitis, gastritis	Lymphadenomegaly, splenomegaly, hepatomegaly	ITP	Mastoiditis	anti-infection prophylaxis, IVIG, glucocorticoid, mTOR inhibitor	Alive
P11	M	3	100	E1021K	Diarrhea	Recurrent respiratory tract infections(1y), bronchiectasis(9y)	Chronic diarrhea, rectocolitis	Lymphadenomegaly, splenomegaly, hepatomegaly	Hypothyroidism, thrombocytopenia	Failure to thrive, nasosinusitis, mastoiditis	anti-infection prophylaxis, IVIG, glucocorticoid, mTOR inhibitor, HSCT	Alive
P12	M	3	47	E1021K	Pneumonia	Recurrent respiratory tract infections(3m)		Lymphadenomegaly, splenomegaly, hepatomegaly	AIHA	Leukoencephalopathy, failure to thrive, mastoiditis, demyelinat	anti-infection prophylaxis, IVIG, glucocorticoid, mTOR inhibitor	Alive
P13	M	24	61	E1021K	Upper respiratory infection	Recurrent respiratory tract infections(2.5y), bronchiectasis		Lymphadenomegaly, splenomegaly, hepatomegaly		Nasosinusitis, mastoiditis	anti-infection prophylaxis, IVIG, mTOR inhibitor	Alive
P14	F	36	50	E1021K	Upper respiratory infection	Recurrent respiratory tract infections(3y), pneumothorax		Lymphadenomegaly, splenomegaly			anti-infection prophylaxis, HSCT	Alive
P15	M	8	60	E1021K	Pneumonia	Recurrent respiratory tract infections(8m), bronchiectasis		Lymphadenomegaly, splenomegaly, hepatomegaly		Urinary tract infection	anti-infection prophylaxis, IVIG, mTOR inhibitor, HSCT	Alive
P16	M	36	169	E1021K	Splenomegaly	Recurrent respiratory tract infections(3y)	Chronic diarrhea, colonic ulcers	Lymphadenomegaly, splenomegaly, hepatomegaly	IBD		anti-infection prophylaxis, IVIG	Alive
P17	M	18	99	E525G	Upper respiratory infection	Recurrent respiratory tract infections(1.5y), bronchiectasis(7y), otitis media, hearing loss		Lymphadenomegaly, splenomegaly, hepatomegaly	ILD	Kidney stones, oblique inguinal hernia, failure to thrive, mastoiditis	anti-infection prophylaxis, IVIG, glucocorticoid, mTOR inhibitor	Alive
P18	F	6	109	E1021K	Upper respiratory infection	Recurrent respiratory tract infections(6m), bronchiectasis(9y), otitis media, hearing loss	Chronic diarrhea, hematochezia	Lymphadenomegaly, splenomegaly	ITP, IBD		anti-infection prophylaxis, IVIG, glucocorticoid, mTOR inhibitor, HSCT	Alive
P19	M	8	108	E1021K	Upper respiratory infection	Recurrent respiratory tract infections(8m), bronchiectasis(12y)	Small intestinal ulcers, proctitis, colitis(1)	Lymphadenomegaly, splenomegaly, hepatomegaly	IBD	Ventricular septal defect, failure to thrive	anti-infection prophylaxis, IVIG, mTOR inhibitor	Alive
P20	M	3	160	E1021K	Upper respiratory infection	Recurrent respiratory tract infections(3m), bronchiectasis(12y), otitis media		Lymphadenomegaly, splenomegaly			anti-infection prophylaxis, IVIG, mTOR inhibitor	Alive
P21	M	3	65	E1021K	Upper respiratory infection	Recurrent respiratory tract infections(3m), otitis media, hearing loss		Lymphadenomegaly, splenomegaly, hepatomegaly			anti-infection prophylaxis, IVIG, mTOR inhibitor	Alive
P22	F	16	21	E1021K	Diarrhea	Recurrent respiratory tract infections(2y), bronchiectasis(6y)	Proctitis, colitis, intestinal obstruction	Lymphadenomegaly, splenomegaly	IBD	Nasosinusitis	anti-infection prophylaxis, IVIG, mTOR inhibitor	Alive
P23	M	18	21	E1021K	Diarrhea	No	Duodenitis, colitis	Lymphadenomegaly, splenomegaly, hepatomegaly	Hypothyroidism, IBD	Dysmyelination	anti-infection prophylaxis, IVIG, glucocorticoid, mTOR inhibitor, HSCT	Alive
P24	F	0.5	65	E1021K	Pneumonia	Recurrent respiratory tract infections(2w), otitis media	Esophagitis, gastritis, duodenitis, colitis, rectitis(1)	Lymphadenomegaly, hepatomegaly	IBD	Atrial septal defect, nasosinusitis, mastoiditis	anti-infection prophylaxis, IVIG, mTOR inhibitor, HSCT	Alive
P25	F	1	179	E1021K	Pneumonia	Recurrent respiratory tract infections(1m), bronchiectasis(14y)		Lymphadenomegaly, splenomegaly, hepatomegaly	SLE, lupus nephritis	Failure to thrive, nasosinusitis	anti-infection prophylaxis, IVIG, glucocorticoid, mTOR inhibitor	Alive
P26	M	29	50	E1021K	Axillary lymph nodes enlargement	Recurrent respiratory tract infections(2.5y), bronchiectasis(4y)		Lymphadenomegaly, splenomegaly, hepatomegaly		Hematuresis	anti-infection prophylaxis, IVIG, mTOR inhibitor	Alive
P27	F	24	88	E1021K	Pneumonia	Recurrent respiratory tract infections(2y), bronchiectasis(7y), otitis media, hearing loss	Colon ulcer, gastritis, enteritis(1)	Lymphadenomegaly, splenomegaly, hepatomegaly	IBD	Hypoalbuminemia, nasosinusitis, mastoiditis	anti-infection prophylaxis, IVIG, mTOR inhibitor	Alive
P28	F	1	62	E1021K	Pneumonia	Recurrent respiratory tract infections(3m), otitis media		Lymphadenomegaly, splenomegaly		BCGitis	anti-infection prophylaxis, IVIG, glucocorticoid, mTOR inhibitor	Alive
P29	F	48	70	E1021K	Pneumonia	Recurrent respiratory tract infections(4m), otitis media		Lymphadenomegaly, splenomegaly, hepatomegaly	Thrombocytopenia	Mastoiditis, arachnoid cyst, dysmyelination, nasosinusitis, mastoiditis	anti-infection prophylaxis, IVIG, mTOR inhibitor	Alive
P30	F	52	57	E1021K	Hematochezia	Recurrent respiratory tract infections(6y)	Hematochezia, gastritis, colitis	Lymphadenomegaly, splenomegaly	IBD	Urinary tract infection, chronic recurrent parotitis, mastoiditis	anti-infection prophylaxis, IVIG, mTOR inhibitor	Alive
P31	M	62	68	E1021K	Abdominal pain	No	Intussusception, peritonitis, intestinal obstruction, acute peritonitis, acute appendicitis, multiple polyps of ileum	Lymphadenomegaly, splenomegaly, hepatomegaly		Inguinal hernia, nasosinusitis	anti-infection prophylaxis, IVIG, mTOR inhibitor	Alive
P32	M	12	116	E1021K	Pneumonia	Recurrent respiratory tract infections(1y), bronchiectasis(10y)	Gastrointestinal bleeding, suppurative appendicitis, coloproctitis, duodenitis	Lymphadenomegaly, splenomegaly, hepatomegaly	IBD	Severe malnutrition, Grade I atrioventricular block, conjunctivitis, hypertension, osteoporosis, failure to thrive, nasosinusitis, mastoiditis	anti-infection prophylaxis, IVIG, glucocorticoid, mTOR inhibitor, HSCT	Died
P33	M	49	52	E1021K	Hematochezia	Recurrent respiratory tract infections(4y)	Chronic gastritis and colitis, duodenitis	Lymphadenomegaly, splenomegaly, hepatomegaly	IBD		anti-infection prophylaxis, IVIG, glucocorticoid, mTOR inhibitor, HSCT	Alive
P34	M	3	120	E1021K	Pneumonia	Recurrent respiratory tract infections(3m), bronchiectasis		Lymphadenomegaly, splenomegaly	Arthritis	Nasosinusitis	anti-infection prophylaxis, IVIG, mTOR inhibitor	Alive
P35	M	24	28	E1021K	Pneumonia	Recurrent respiratory tract infections(2y), bronchiectasis	Chronic diarrhea, small intestinal nested	Lymphadenomegaly, splenomegaly		Inguinal hernia, nasosinusitis, mastoiditis	anti-infection prophylaxis, IVIG, glucocorticoid	Alive
P36	M	48	67	E1021K	Pneumonia	Recurrent respiratory tract infections(4y), otitis media	Chronic diarrhea, gastritis, duodenal bulb inflammation, colitis, rectitis	Lymphadenomegaly, splenomegaly, hepatomegaly	Thrombocytopenia, IBD	Urinary tract infection, failure to thrive, nasosinusitis, mastoiditis	anti-infection prophylaxis, IVIG, glucocorticoid, mTOR inhibitor	Alive
P37	F	38	41	E1021K	Upper respiratory infection	Recurrent respiratory tract infections(3y), otitis media		Lymphadenomegaly, splenomegaly, hepatomegaly		Osteomyelitis, contact dermatitis, mastoiditis	anti-infection prophylaxis, IVIG, mTOR inhibitor	Alive
P38	F	48	64	E1021K	Submaxillary lymph node enlargement	Otitis media		Lymphadenomegaly, splenomegaly, hepatomegaly		Nasosinusitis, failure to thrive, nasosinusitis, mastoiditis	anti-infection prophylaxis, glucocorticoid, mTOR inhibitor	Alive
P39	M	6	48	E1021K	Upper respiratory infection	Recurrent respiratory tract infections(6m), bronchiectasis(4.5y)		Lymphadenomegaly, splenomegaly, hepatomegaly		Osteomyelitis, nasosinusitis, thick corpus callosum	anti-infection prophylaxis, IVIG, glucocorticoid, mTOR inhibitor	Alive
P40	F	100	103	E1021K	Purpura			Lymphadenomegaly, splenomegaly, hepatomegaly	Cutaneous vasculitis	Nasosinusitis, mastoiditis	anti-infection prophylaxis, IVIG, mTOR inhibitor	Alive
P41	M	3	108	E1021K	Purpura	Recurrent respiratory tract infections(6y), bronchiectasis(8y), otitis media	Hematochezia	Lymphadenomegaly, splenomegaly, hepatomegaly	ITP	Failure to thrive	anti-infection prophylaxis, IVIG, glucocorticoid, mTOR inhibitor	Alive
P42	M	84	105	E1021K	Abdominal pain	Recurrent respiratory tract infections(3y), otitis media		Lymphadenomegaly		Nasosinusitis	anti-infection prophylaxis, IVIG, mTOR inhibitor	Alive

(1) Detected by gastrointestinal endoscopy, without clinical presentation; Y, years; M, male; F, female; PAH, pulmonary artery hypertension; SLE, systemic lupus erythematosus; IBD, inflammatory bowel disease; ILD, interstitial lung disease; ITP, immune thrombocytopenic purpura; AIHA, autoimmune hemolytic anemia; IVIG, intravenous immunoglobulin; hematopoietic stem cell transplantation.

### Immunological Features

The immunological characteristics of the patients are listed in Table 2, Table S1 and Table S2. Thirty-four patients (81%) in our cohort presented with elevated IgM levels (median = 3.7 g/L, IQR = 2.1–5.3 g/L) at the time of diagnosis. Decreased IgG levels (median = 8.7 g/L, IQR = 3.8–14.3 g/L) were detected in 12 patients (29%) before treatment, while 11 patients (26%) had elevated IgG levels. Thirty-seven patients (90%) had normal IgE levels, with 4 patients (10%) having elevated IgE levels. Fifteen patients (36%) had decreased serum IgA levels (median = 0.68 g/L, IQR = 0.2–1.2 g/L), while one patient with anaphylactoid purpura (P40) had elevated serum IgA levels. Hepatitis B surface antibodies were detected in 39 patients.

**Table 2:** Immunological characteristics of APDS1 patients

	Decreased, *n*/total (%)	Normal, *n*/total (%)	Increased, *n*/total (%)
CD3+ T cells	20/42 (48)	7/42 (17)	15/42 (35)
CD3+ T cell counts	29/42 (69)	8/42 (19)	5/42 (12)
CD4+ T cells	32/42 (76)	7/42 (17)	3/42 (7)
CD4+ T cell counts	35/42(83)	7/42 (17)	0/42 (0)
CD8+ T cells	7/42 (17)	10/42 (24)	25/42 (59)
CD8+ T cell counts	16/42(38)	20/42 (48)	6/42 (14)
CD19+ B cells	32/42 (76)	7/42 (17)	3/42 (7)
CD19+ B cell counts	34/42 (81)	8/42 (19)	0/42 (0)
CD16CD56+ NK cells	7/42 (17)	16/42 (39)	19/42 (45)
CD16CD56+ NK cell counts	16/42 (38)	17/42 (41)	9/42 (21)
CD4/CD8	33/42 (79)	9/42 (21)	0/42 (0)
αβDNT	4/14 (29)	10/14 (71)	0/14 (0)
γδT	9/14 (64)	5/14 (36)	0/14 (0)
CD4 NAÏVE	28/29 (97)	1/29 (3)	0/29 (0)
CD4 CM	0/29 (0)	12/29 (41)	17/29 (59)
CD4 EM	0/29 (0)	4/29 (14)	25/29 (86)
CD4 TEMRA	1/15 (7)	9/15 (60)	5/15 (33)
CD8 NAÏVE	28/29 (97)	1/29 (3)	0/29 (0)
CD8 CM	0/29 (0)	20/29 (69)	9/29 (31)
CD8 EM	0/29 (0)	5/29 (17)	24/29 (83)
CD8 TEMRA	0/29 (0)	18/29 (62)	11/29 (38)
Naïve B	10/29 (34)	14/29 (48)	5/29 (17)
Memory B	11/29 (40)	13/29 (45)	5/29 (17)
Transitional B	0/29 (0)	3/29 (10)	26/29 (90)
Marginal zone B	7/14 (50)	7/14 (50)	0/14 (0)
Plasmablasts	0/29 (0)	14/29 (48)	15/29 (52)
Immunoglobulin			
IgG	12/42 (29)	19/42 (45)	11/42 (26)
IgA	15/42 (36)	26/42 (62)	1/42 (2)
IgM	0/42 (0)	8/42 (19)	34/42 (81)
IgE	0/41 (0)	37/41 (90)	4/41 (10)

αβDNT, double negtive T cell(CD3+CD4-CD8-TCRαβ+); γδT(CD3+TCRγδ+); NAÏVE, naive T cells (CD27+CD45RA+); CM, central memory (CD27+CD45RA-); EM, effector memory (CD27-CD45RA-); TEMRA, terminally differentiated effector memory T cells (CD27-CD45RA+); Naive B(CD19+CD27-IgD+); Memory B(CD19+CD27+); Marginal zone B(CD19+CD27+IgD+); Plasmablasts(CD19+CD38high+); Transitional B(CD19+CD38high+CD24high+).

The lymphocyte repertoire in APDS1 patients can be variable, but some common features are shared in these patients. Sixteen patients (38%) presented with normal proportions of natural killer (NK) cells, whereas 19 patients (45%) presented with increased proportions of NK cells. However, 16 patients (38%) presented with a decreased NK cell count. The proportion and count of T cells were reduced by 48% and 69%, respectively, in APDS1 patients (median = 1040.2 cells/µl, IQR = 731.9–1529.1 cells/µl). A reduced proportion and count of CD4+ T cells were observed in 76% and 83% of patients, respectively (median = 385.0 cells/µl, IQR = 265.6–503.8 cells/µl). Among the CD4+ T-cell subsets, reduced proportions of naïve CD4+ T cells (28/29, 97%) and increased proportions of CD4+ effector memory cells (25/29, 86%) and central memory cells (17/29, 59%) were observed in most of the patients. Increased proportions of CD8+ T cells were found in 59.5% of patients, but the counts were normal in 48% of patients (median = 558.0 cells/µl, IQR = 380.3–903.3 cells/µl). The pathogenic changes in CD8+ T cells were consistent with those in CD4+ T cells and included a decrease in naïve T cells (28/29, 97%) and an increase in effector memory cells (24/29, 83%), central memory cells (9/29, 31%) and terminally differentiated effector memory T cells (11/29, 38%). A reduction in B-cell counts was remarkable in APDS1 patients (median = 160.0 cells/µl, IQR = 66.1–256.3 cells/µl). The frequency of memory B cells was decreased in 11 of 29 (38%) patients. The proportions of transitional B cells were increased in 26 of 29 (90%) patients, and the proportions of plasmablast cells were increased in 15 of 29 (52%) patients, whereas the proportions of naïve B cells were decreased in 10 of 29 (34%) patients. Marginal zone B cell proportions were also decreased in 7 of 14 (50%) patients.

We further divided patients with autoimmunity into an AD group (patients with autoimmunity), and we assigned the other patients to the NAD group (patients without autoimmunity). We compared the immunological examination results between these groups. We found that the median CD3+ T-cell count was 854 cells/µl in the AD group, which was lower than that in the NAD group (1302 cells/µl, *p* = 0.039). The median B-cell count was 91.97 cells/µl in the AD group, which was lower than that in the NAD group (248.33 cells/µl, *p* = 0.003). Additionally, the IgG level was greater in the AD group than in the NAD group. The statistical results are shown in Table S3.

### Deep Immunophenotyping

**Fig. 2:**
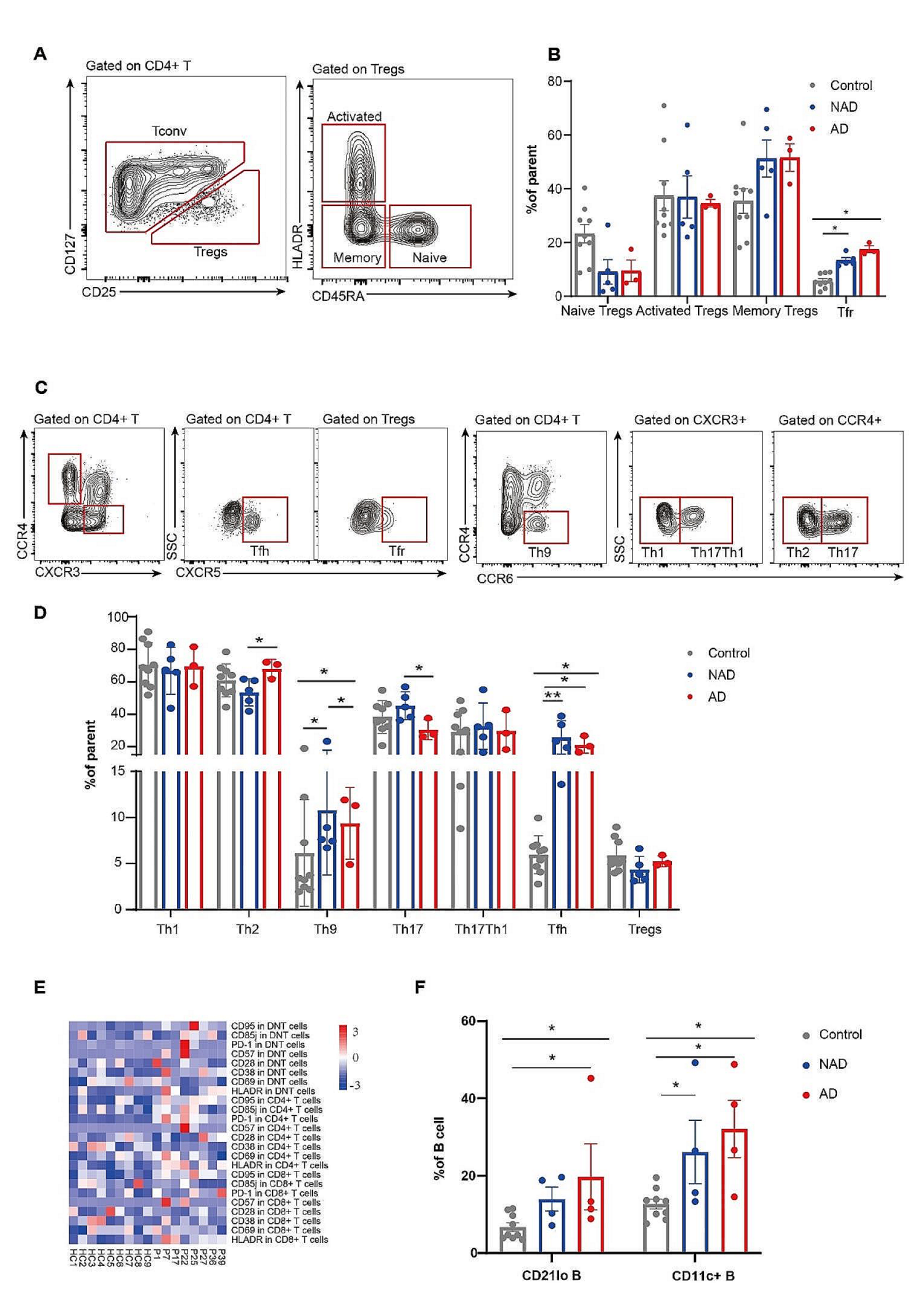
Immune cell subsets distribution in the peripheral blood of APDS patients. **(A)** Gating strategy for the analysis of Tregs. **(B)** Percentages of Tfr(CD4+CD25-CD127-CXCR5+), naïve Tregs (CD4+CD25-CD127-CD45RA+HLADR-), activated Tregs (CD4+CD25-CD127-CD45RA-HLADR+) and memory Tregs (CD4+CD25-CD127-CD45RA-HLADR-) in healthy controls and APDS1 patients. **(C)** Gating strategy for the analysis of T helper cells and Tfr. **(D)** Proportions of Th1 (CD4+CXCR3+CCR4-CCR6-), Th1-like Th17 (CD4+CXCR3+CCR4-CCR6+), Th2 (CD4+CXCR3-CCR4+CCR6-), Th9 (CD4+CCR4-CCR6+), Th17 (CD4+CXCR3-CCR4+CCR6+), Tfh (CD4+CXCR5+), and Tregs (CD4+CD25-CD127-) in controls and the patients. **(E)** MFI of different markers in different T cell subsets. **(F)** Percentages of CD11c+ B cells (CD19+CD11c+) and CD21lo B cells (CD19+CD21lo) in healthy controls and APDS1 patients. AD, patients with autoimmunity; NAD, patients without autoimmunity. *p < 0.05; **p < 0.01. Bars indicate mean ± SD

To further elucidate the immunophenotypes of the APDS1 patients, we characterized the immune cells through multiparametric flow cytometry, and the results are shown in Fig. 2, Table S4, Table S5 and Table S6. Fresh whole-blood samples from 8 patients with 2 different *PIK3CD* mutations (patients 1, 7, 22, 25, 27, 36, and 39 with E1021K; patient 17 with E525G) and 9 controls were further analyzed to determine the proportions of immune cell subsets. At the time of the blood sampling, 3 patients (P17, P25, and P36) had autoimmune manifestations. As shown in Fig. 2D, the proportion of regulatory T cells (Tregs) in the NAD group and AD group was lower than that in the control group, but the difference was not significant. We found that the proportion of naïve Tregs was lower in patients than in controls (Fig. 2B). Among the APDS1 patients, the proportion of T helper 2 (Th2) cells was greater in the AD group than in the NAD group (*p* < 0.05, Fig. 2D); the proportion of T helper 9 (Th9) cells differed among the control, AD, and NAD groups (*p* < 0.05, Fig. 2D); and the proportion of T helper 17 (Th17) cells in the AD group was lower than that in the control and NAD groups (*p* < 0.05, Fig. 2D). The proportion of T follicular helper (Tfh) cells in APDS1 patients was greater than that in controls (*p* < 0.05, Fig. 2D), but there was no significant difference between the AD group and the NAD group. APDS1 patients also had a greater proportion of T follicular regulatory (Tfr) cells (*p* < 0.05, Fig. 2B), and the mean value in the AD group was greater than that in the NAD group, but the difference was not significant. High-throughput flow cytometry revealed a greater mean fluorescence intensity (MFI) of PD1 in CD4+ T cells and CD8+ T cells (*p* < 0.05; Fig. 2E). In addition, the expression of HLA-DR on the CD4+ T-cell and CD8+ cell surfaces were greater in APDS1 patients than in controls (*p* < 0.01; Fig. 2E), indicating that T cells in patients are in the late stage of activation. Moreover, CD95 was highly expressed in CD4+ T cells, CD8+ T cells and double-negative T (DNT) cells (*p* < 0.01; Fig. 2E). The proportion of CD11c+ B cells was increased in both the AD and NAD groups compared with the control group (*p* < 0.05, Fig. 2F). The proportion of CD21lo B cells was increased in APDS1 patients (*p* < 0.05, Fig. 2F), and the proportion was greater in the AD group than in the NAD group.

### Autoantibody Analysis

**Fig. 3:**
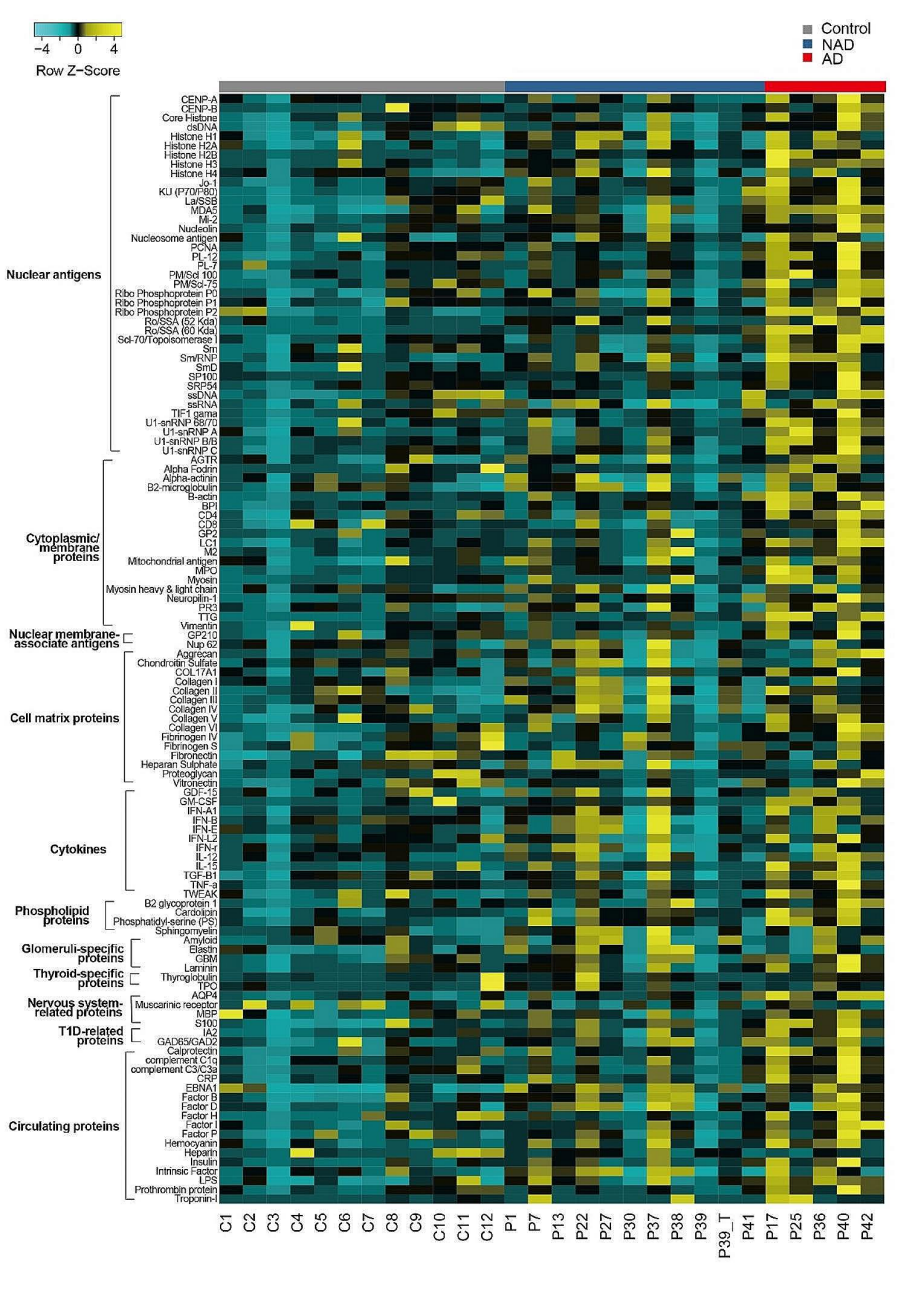
Antigen array profiling identifies a different IgG autoantibody activity in APDS1 patients. Antigens were classified in to nuclear antigens, cytoplasmic/membrane proteins, nuclear membrane associate antigens, cell matrix proteins, cytokines, phospholipid proteins, glomeruli-specific proteins, thyroid-specific proteins, nervous system related proteins, T1D-related proteins, and circulating proteins. Heatmap displaying NFI after Z-score normalization. Yellow represents high reactivity, black intermediate reactivity, and cyan lack of reactivity. Gray represents controls, blue represents patients without autoimmunity, and red represents patients with autoimmunity

**Fig. 4:**
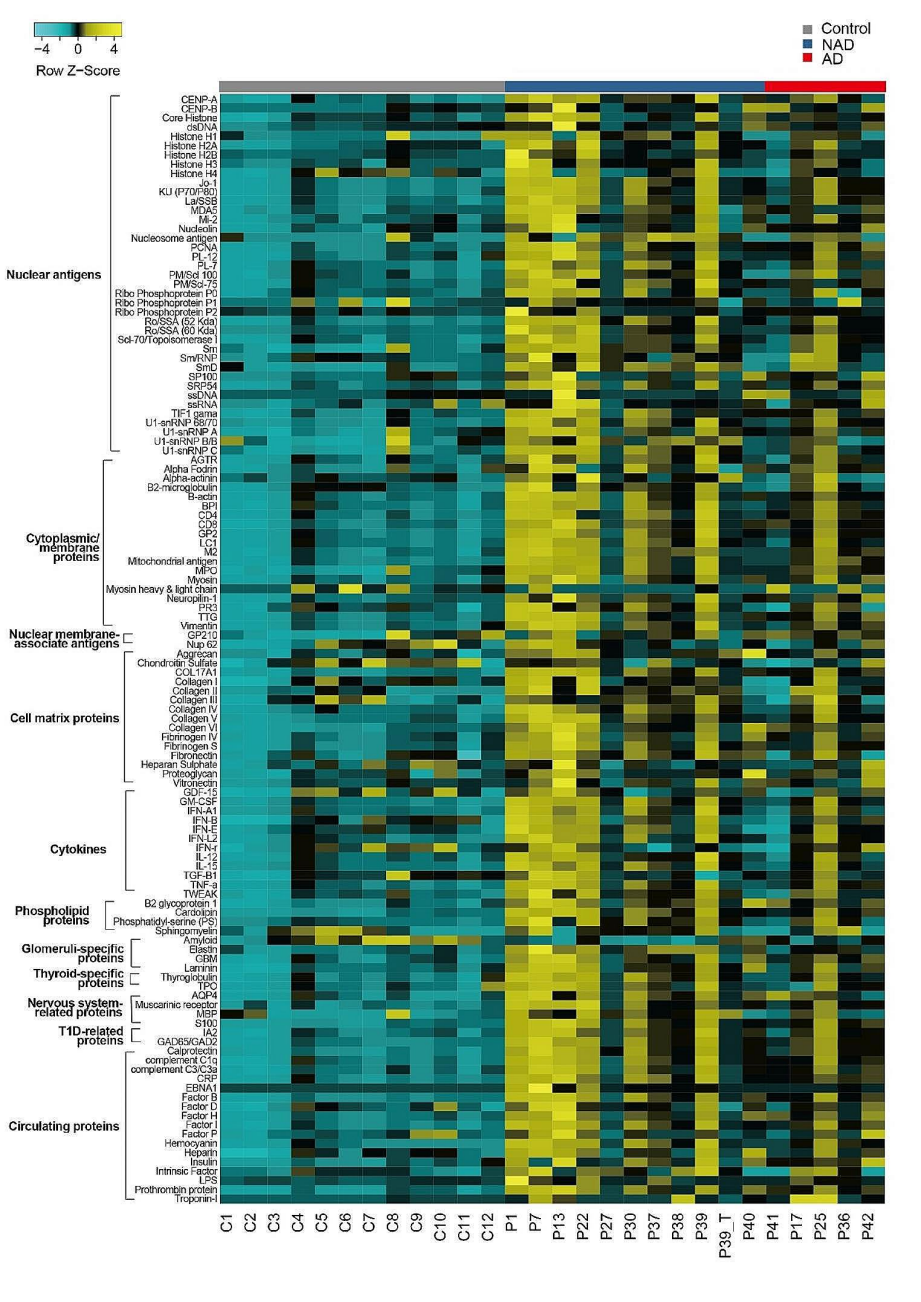
Antigen array profiling identifies a different IgM autoantibody activity in APDS1 patients. Heatmap displaying NFI after Z-score normalization. Yellow represents high reactivity, black intermediate reactivity, and cyan lack of reactivity. Gray represents controls, blue represents patients without autoimmunity, and red represents patients with autoimmunity

We tested 16 serum samples from 15 patients in which serum antibody titers for P39 were measured 3 months before and after systemic treatment. Specific information can be found in Table S5. Among these fifteen patients, 5 had obvious autoimmune manifestations or were positive for autoantibodies at the time of autoantibody testing, including P17, P25, P36, P40, and P42. Over 120 proteins representing autoantigen epitopes were printed on the autoantigen microarray to determine the IgM/IgG antibody reactivity in serum. The NFI values of the IgG and IgM autoantibodies are shown in Figs. 3 and 4. In a comparison of the three groups, a total of 47 antigen and IgG antibody signal values were significantly different (*p* < 0.05). Among all the antibodies with differences, 12 IgG antibodies against Jo-1, SSB, PL-12, PM/scl100, Scl-70, TIF1γ, β-actin, MPO, TTG, AQP4, calprotectin, and CRP were more abundant in patients with autoimmunity than in those without (*p* < 0.05). Figure 3 shows the levels of all IgG antibodies against self-antigens in patients and controls. Patients with autoimmunity had high signal values for multiple autoantibodies. Two patients (P22 and P37) were previously considered to have IBD. After treatment with immunosuppressants, their condition improved, and there were no signs of inflammation, but high levels of multiple antibodies were still found. The difference in IgM antibodies was more significant between controls and patients (Fig. 4), with a total of 102 items showing significant differences (*p* < 0.05). However, there was no significant difference in any IgM antibody between the AD group and the NAD group. As shown in Fig. 4, almost all the IgM autoantibodies in the NAD group showed high signal values. We compared the serum IgM levels between the AD and NAD groups and found that the IgM level was greater in the NAD group (Fig. S1). We retested one patient (P39) for serum antibodies after 3 months of regular intravenous immunoglobulin (IVIG) and sirolimus treatment. This patient achieved marked recovery in terms of both the proportion and the number of immune cells (Table S8). Unexpectedly, the NFI of almost all the IgG autoantibodies was increased.

**Fig. 5:**
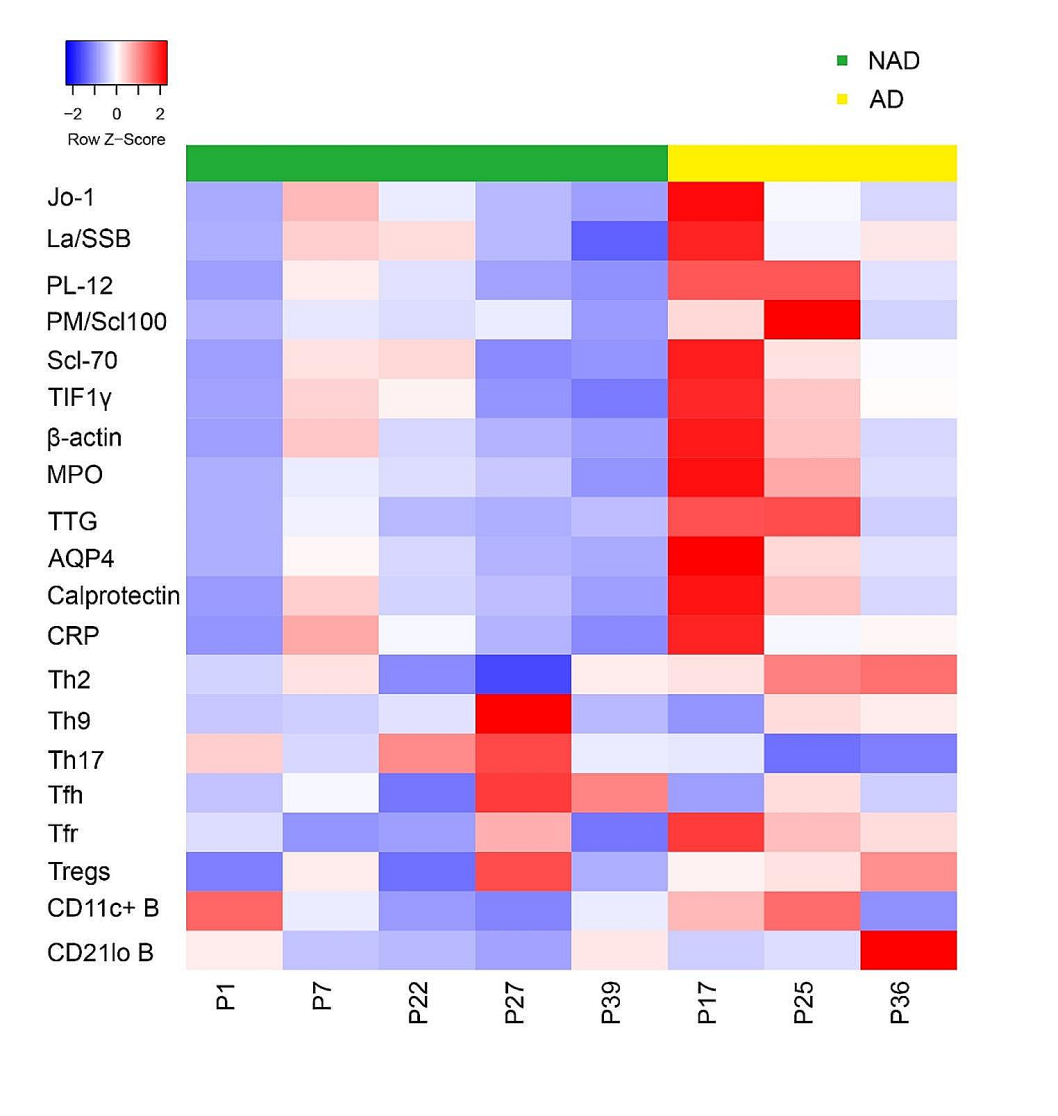
Analysis of the correlation between differential immune cell autoantibodies in APDS1 patients. Green represents patients without autoimmunity, and yellow represents patients with autoimmunity

To further explore the connection between autoantibodies and immune cells, we compared 8 patients who underwent deep immunophenotyping and protein array analysis (Fig. 5). In patients with autoimmunity, the proportion of Tfr cells was greater, and the proportion of Tfr cells might be positively correlated with autoantibody levels. The proportion of Tregs was lower in APDS1 patients than in controls. A comparison of 3 patients with autoimmunity revealed that the levels of autoantibodies and proportion of Tregs might be negatively correlated. Furthermore, in APDS1 patients with autoimmunity, the proportion of Th2 cells may be negatively correlated with autoantibody levels.

## Discussion

We identified 42 Chinese patients with mutations in the *PIK3CD* gene whose clinical phenotype and immunological features were similar to those described earlier [5]. As previously reported, recurrent respiratory tract infection is the most common clinical phenotype [5, 19–21]. In our cohort, 88% of patients developed respiratory infections at an early age, and 60% of patients presented with respiratory infections as the first symptom. Bronchiectasis was detected in 59% patients with recurrent respiratory tract infections, which is a much greater percentage than that in previous studies [22]. Previous studies have shown that patients with bronchiectasis have normal IgG levels [21], but this was not found in our cohort. In the present study, magnetic resonance imaging (MRI) suggested that 45% of patients had nasosinusitis and 40% of patients had mastoiditis, which are often ignored.

In past reports, the incidence of autoimmunity ranged from 28–68% [12, 21, 22]. In this study, 55% of the patients were considered to have autoimmunity, but some patients lacked a clear diagnostic basis, which hindered the diagnosis and effective intervention of autoimmunity. In the past, through studies on APDS patients and animal models, it was found that the loss of self-tolerance of B cells, cross-reaction between B cells and commensal bacteria, and excessive differentiation of Tfh cells may be important mechanisms for the occurrence of autoimmunity [14, 23].

In the present study, we found that the number of CD3+ T cells and B cells were lower in patients with autoimmunity. In past studies, it was found that T cells and B cells are prone to apoptosis [5, 24]. Additionally, hyperactive PI3K affected B cell development and differentiation in the bone marrow and the periphery [25, 26]. We speculated that apoptosis increases in patients with autoimmunity, resulting in a more obvious decrease in T cells and B cells. Th2 cells promote B-cell differentiation by secreting cytokines such as IL-4, IL-6, and IL-10, which increase the occurrence of SLE [27]. However, in patients with APDS1 with autoimmunity, the proportion of Th2 cells was increased and inversely correlated with autoantibody levels. Further study is needed to determine the pathogenic mechanism of action of Th2 cells in APDS1 patients. We found that the proportion of Th9 cells was increased in APDS1 patients. Previous studies have revealed that Th9 cells may mediate the occurrence of allergies and IBD, which may partially explain the high incidence of intestinal inflammation in APDS1 patients [28]. Tfh cells play a vital role in the selection and differentiation of B cells in germinal center, but an elevated Tfh cell proportion also contributes to autoimmune disease [14, 29, 30]. In APDS1 patients, the proportion of Tfh cells has been shown to be significantly increased [14, 31, 32], and this may also contribute to autoimmunity. Interestingly, although the proportion of Tregs was decreased in APDS1 patients, the proportion of Tfr cells was increased, especially in patients with autoimmunity. In general, the proportions of Tregs and Tfr decreases, and the proportion of Tfh increases in autoimmune diseases [33, 34], but there were different changes in APDS1 patients. Some scholars have found that the proportion of Tfr in patients with SLE is increased and is positively correlated with the level of autoantibodies and the severity of the disease [35]. An increase in the proportion of Tfr cells may indicate that the patient is in a severe autoimmune state. However, due to the small sample size, it is unclear whether the changes of Tfr cells are associated with disease severity. Further research should be conducted on whether *PI3KCD* gene defects affect Tfr cell function. Moreover, deep immunophenotyping revealed that the populations of CD21lo B cells and CD11c+ B cells were increased; these cells are defined as autoreactive B cells and are strongly associated with autoimmune diseases [36–39]. Previous studies have revealed that IgM antibodies are mainly produced by B1 cells in murine and CD21lo B cells in human [40]. Previously, studies have found an increase in CD21lo B cells in common variable immunodeficiency (CVID) patients, including patients with *PIK3CD* gene mutation [41]. The changes in these immune cells caused by excessive activation of the PI3K signaling pathway may be the pathogenic factor of autoimmunity in APDS1 patients.

The present study compared serum autoantibody levels in APDS1 patients and revealed that autoimmune patients had a wide range of IgG autoantibodies, which was consistent with previous findings in mouse models [14]. Furthermore, many autoantibodies were found in 2 patients without clinically diagnosed autoimmunity. These two patients were previously considered to have IBD, but their conditions improved after treatment, and they had no signs of autoimmunity. In the autoimmune patient group, there were significant differences in antibody titers against 12 autoantigens, among which Jo-1, SSB, PL-12, PM/Scl100, Scl-70, TIF1γ, and MPO were associated with rheumatic diseases [42], TTG was associated with intestinal inflammatory diseases [43], and AQP4 was associated with demyelinating diseases [44]. Given the high incidence of neurological abnormalities and IBD in patients with APDS1, researchers and clinicians must pay attention to whether these antibodies play a pathogenic role. Notably, one patient’s symptoms improved after treatment, but the level of IgG autoantibodies increased, and no autoimmune manifestations were found during follow-up. The expansion of autoreactive B cells after treatment should be considered to avoid effects in other organ systems. Since the clinical diagnosis of autoimmunity is based on a combination of clinical phenotypes and related laboratory test findings, autoantigen chips can be used to detect the presence of autoantibodies early, to help clinicians intervene early in autoimmune phenomena, and to evaluate the effectiveness of treatment. We also found that APDS1 patients had increased levels of IgM autoantibodies, which were correlated with serum IgM levels. This finding suggests that most of the IgM antibodies in APDS1 patients respond to self-antigens, and this finding is consistent with previous reports [23]. IgM clones that react to self-antigens have been identified [45–47]. However, further exploration is needed to determine whether excessive IgM autoantibody levels increase the occurrence of autoimmunity in APDS1 patients.

In conclusion, we reported the clinical manifestations of 42 Chinese patients with *PIK3CD* mutations. Recurrent respiratory tract infections were the most significant clinical manifestations in APDS1 patients. Autoimmune status was common but also difficult to identify in APDS1 patients. Lower number of CD3+ T cells and B cells and higher IgG levels were reported in patients with autoimmunity. The imbalance in the proportion of subsets of cells that may contribute to autoimmunity includes a decrease in Tregs and an increase in the proportions of Th9, Tfh, CD11c+ B and CD21lo B cells. Autoantibody testing revealed a wide range of IgG/IgM autoantibodies in APDS1 patients. We found there might be a positive correlation between the proportion of Tfr and autoantibody levels in autoimmune patients. There are some limitations to this research. Our research is based on a small group of patients, and further research should be performed to better understand the mechanism of autoimmunity in APDS1 patients.

### Electronic Supplementary Material

Below is the link to the electronic supplementary material.

Supplementary Material 1

Supplementary Material 2

Supplementary Material 3

Supplementary Material 4

Supplementary Material 5

Supplementary Material 6

Supplementary Material 7

Supplementary Material 8

Supplementary Material 9

Supplementary Material 10

Supplementary Material 11

Supplementary Material 12

## Data Availability

The data used or analyzed during the current study is provided within the manuscript and supplementary information files and are available from the corresponding author upon reasonable request.
