# Supplementary material for: Phenotypic and Immunological Characterization of Patients with Activated PI3Kδ Syndrome 1 Presenting with Autoimmunity
Source: J Clin Immunol. 2024 Apr 18;44(4):102. doi: 10.1007/s10875-024-01705-w (PMC11026262; doi:10.1007/s10875-024-01705-w)

Table S1. Immunological tests of APDS1 patients

ID	Age	Sex	CD16CD56%	CD16CD56 cells/ul	CD3%	CD3 cells/ul	CD19%	CD19 cells/ul	CD4%	CD4 cells/ul	CD8%	CD8 cells/ul	CD4/CD8	IGG g/L	IGA g/L	IGM g/L	IGE g/L	HBsAb(mIU/mL)
P1	11Y	M	61.42↑	2798.15↑	33.89↓	1543.9	4.29↓	195.56↓	8.91↓	405.83↓	24.25	1105	0.37↓	1.55↓	0.99	15.31↑	13.9	46
P2	11Y	F	37.97↑	552.84	56.34↓	820.27↓	3.6↓	52.39↓	22.5↓	327.64↓	26.75	389.41↓	0.84↓	4.95↓	2.53	43.2↑	23.8	46
P3	11Y	M	4.2↓	147↓	92.39↑	3230↑	3↓	109↓	11↓	386↓	80↑	2800↑	0.14↓	0.1	6.76	1.8	0	97
P4	14Y	M	9.96↓	355.41	86.75↑	3096.83↑	1.16↓	41.38↓	19.76↓	705.51	66↑	2356.31↑	0.3↓	7.47	0.39↓	4.2↑	207.8↑	59
P5	3Y	M	17.12	1961↑	73.55↑	8427↑	8.74↓	1001	12.63↓	1447	47.63↑	5457↑	0.27↓	1.38↓	0.54	2.47↑	26	97
P6	5Y	F	12.03	108.11↓	83↑	745.8↓	3.97↓	35.71↓	23.57↓	211.75↓	51.79↑	465.41↓	0.46↓	4.7↓	0.29↓	10.66↑	36.9	125
P7	7Y	F	41.43↑	861.09↑	50.64↓	1052.6	7.36↓	152.94↓	24.17↓	502.35↓	26.02	504.9↓	0.93↓	11.3	1.21	3.84↑	16.9	62
P8	5M	M	10.8↓	279.34	53.57↓	1385.3↓	34.74↑	898.3	30.88	798.44↓	21.52↓	556.49	1.43↓	5.26	0.15	1.77↑	15.4	0↓
P9	5Y	M	11	190↓	76↑	1302↓	12↓	210↓	22↓	384↓	51↑	881	0.44↓	19↑	1.54	3.8↑	30	250
P10	8Y	M	28.63↑	360	65.64	825.3↓	5.2↓	65.33↓	14.53↓	182.67↓	48.25↑	606.67	0.3↓	9.3	0.99	5.25↑	8.4	27
P11	10Y	M	15.25	67.45↓	74.24↑	328.3↓	9.15↓	40.48↓	27.46↓	121.44↓	44.75↑	191.9↓	0.61↓	3.95↓	1	5.6↑	18.25	230
P12	5Y	M	32.07↑	401.04	55.2↓	690.3↓	12.36↓	154.6↓	21.79↓	272.46↓	32.93	411.75↓	0.66↓	17.4↑	1.21	2.6↑	20.21	460
P13	6Y	M	7.05↓	102.3↓	86.56↑	1253.1↓	6.17↓	89.28↓	19.6↓	283.78↓	55.4↑	801.91	0.35↓	2.9↓	0.14↓	6.01↑	6.95	65
P14	5Y	F	18.17	275.47	63.86↓	968.1↓	16.93	256.58↓	21.5↓	325.85↓	41.02↑	621.78	0.52↓	9.2	0.95	3.1↑	56.52	＞1000
P15	6Y	M	17.04	279↓	70.94	1161.4↓	10.88↓	178.16↓	26.08↓	426.91↓	31.11	509.27↓	0.84↓	10.6	0.54	3.34↑	5.5	69
P16	17Y	M	19.05	674.94	73.46↑	2602.7↑	6.82↓	241.5	14.34↓	508.12↓	51.99↑	1841.95↑	0.28↓	9.8	0.41↓	27.5↑	44.5	177
P17	9Y	M	16.47	190.1↓	77.59↑	895.5↓	5.31↓	61.33↓	35.01	404.1↓	39.15↑	451.89↓	0.89	14.5↑	0.7	2.71↑	30.64	80
P18	9Y	F	14.99	294.41	78.09↑	1533.7	6.18↓	121.35↓	39.47↑	775.15	36.46↑	716.13	1.08	12.3	0.18↓	5.15↑	8.57	126
P19	9Y	M	12.07	405.39	85.93↑	2886.6↑	1.18↓	39.72↓	14.31↓	480.76↓	53.82↑	1807.94↑	0.27↓	14.3↑	0.35↓	7.15↑	635.48↑	84

P20	14Y	M	29.62↑	729.75	53.58↓	1320.2	14.79	364.39	22.5↓	554.38	30.52	752.16	0.74	8.3	0.66	4.74↑	38.2	81
P21	5Y	M	8.23↓	208.15↓	66.73	1688.5	24.38↑	616.96	24.42↓	617.79↓	38.04↑	962.48	0.64↓	7.2	0.02↓	4.93↑	7.41	235
P22	2Y	F	25.65↑	1351.67↑	60.19↓	3171.7	12.65↓	666.76	18.81↓	991.07	40.43↑	2130.69↑	0.47↓	1.5↓	0.01↓	2.83↑	16.95	935
P23	2Y	M	26.27↑	360.07	62.61↓	858.2↓	8.81↓	120.7↓	32.87	450.59↓	29.18	399.96	1.13	1.3↓	0.01↓	11.55↑	92.73	338
P24	6Y	F	17.89	145.71↓	72.83	590.3↓	8.19↓	66.38↓	36.12↑	292.72↓	31.04	251.6↓	1.16	6.6	0.23↓	1.18	53.93	17
P25	17Y	F	12.49	87.96↓	73.27↑	516↓	13.06↓	91.97↓	33.85	238.39↓	37.39↑	263.31↓	0.91↓	17.3↑	1.13	4.13↑	49.3	0.2↓
P26	4Y	M	31.5↑	741.75	41.47↓	976.4↓	25.98↑	611.8	17↓	400.2↓	23.76↓	559.48	0.72↓	6.6	0.15↓	0.94	4.43	19.8
P27	7Y	F	55.51↑	731.5↑	35.62↓	469.3↓	6.47↓	85.29↓	10.99↓	144.86↓	23.08↓	304.15↓	0.48↓	10.7	0.99	3.22↑	17.07	0↓
P28	6Y	F	15.79	402.98	73.05↑	1865	10.2↓	260.51↓	34.97	892.8	34.07↑	869.73	1.03	17.3↑	1.31	3.68↑	39.31	138
P29	6Y	F	39.57↑	1069.87↑	53.04↓	1433.9↓	6.93↓	187.35↓	12.86↓	347.58↓	38.24↑	1033.71	0.34↓	2.9↓	0.02↓	8.3↑	28.47	147
P30	5Y	F	19.31	160.31↓	72.19	599.2↓	6.9↓	57.3↓	28.13↓	233.51↓	40.66↑	337.51↓	0.69↓	9.9	1.09	4.83↑	36.79	187
P31	6Y	M	62.89↑	2205.5↑	29.31↓	1027.8↓	7.3↓	256.15↓	8.39↓	294.41↓	19.1↓	669.88	0.44↓	0.12↓	1.5	3.62↑		106
P32	13Y	M	8.03↓	45.23↓	90.65↑	510.3↓	0.88↓	4.95↓	35.81	201.6↓	45.96↑	258.75↓	0.78	16.3↑	0.21↓	1.53	14.16	268
P33	5Y	M	28.73↑	591.79	60.02↓	1236.3↓	10.63↓	218.89↓	22.85↓	470.58↓	34.06↑	701.59	0.67↓	10.8	1.11	1.9	44.41	181
P34	10Y	M	24.26↑	437.29	62.82↓	1132.5↓	12.01↓	216.56	23.47↓	423.08↓	36.99↑	666.81	0.63↓	20.4↑	1.22	1.85	22.36	＞1000
P35	2Y	M	16.2	221.81↓	69.17	947.2↓	12.21↓	167.18↓	26.72↓	365.82↓	38.71↑	530.11	0.69↓	2.1↓	0.19	1.76	0	285
P36	9Y	M	28.32↑	226.29↓	56.06↓	447.9↓	14.48	115.67↓	20.67↓	165.15↓	29.86	238.58↓	0.69↓	7.2	0.33↓	2.1↑	37.28	205
P37	4Y	F	24.49↑	657.33	55.77↓	1497↓	18.72	502.37↓	12.78↓	342.93↓	39.54↑	1061.24	0.32↓	3.2↓	0.12↓	3.81↑	41.04	＞1000
P38	6Y	F	14.81	184.43↓	63.32↓	788.7↓	19.94	248.33↓	34.69	432.1↓	28.42	352.96↓	1.22	14.6↑	0.91	3.66↑	117.66↑	150
P39	5Y	M	31.55↑	743.31↑	64	1527.5	2.72↓	64.04↓	39↑	918.79	22.35↓	526.47	1.75	9.1	1.59	8.4↑	40.63	98.78

P40	8Y	F	10.77↓	113↓	81↑	854↓	6.85↓	72↓	27↓	287↓	49↑	522	0.55↓	24 ↑	2.5 ↑	1.97	333.45 ↑	41.87
P41	11Y	M	42.74↑	640↑	36.63↓	549↓	19.8	296↓	16.37↓	245↓	19.4↓	290↓	0.84↓	14.3↑	1.21	2.47↑	9.22	55.88
P42	8Y	M	32↑	323	49.5↓	502↓	17	180↓	16.5↓	167↓	21↓	212↓	0.79↓	7.9	0.33	2.15↑	67	240

Y, years; M, male; F, female.

Table S2. Immunophenotyping of peripheral blood lymphocyte subsets in APDS1 patients

	Sex	Age	DNT	αβDNT	γδT	CD4 NAÏVE	CD4 CM	CD4 EM	CD4 TEMRA	CD8 NAÏVE	CD8 CM	CD8 EM	CD8 TEMRA	Naïve B	MB	MZB	PB	TRB
P1	M	11Y	3.3			10.3↓	48.7	41↑		17↓	32.7	28.1↑	22.1	71.9	10.7		8.5	50.8↑
P2	F	11Y	6.1			9.3↓	28.7	34.1↑		20.2↓	33.3	34↑	12.5	5.7↓	31.3↑		23.9↑	3.9
P3	M	11Y	3.2			18.4↓	71.8↑	8.6		10.9↓	16.2	31.9↑	40.9↑	77.8	3.3↓		9.6↑	66.1↑
P5	M	3Y	17.4			14.6↓	63.9↑	34.1↑		20.2↓	23.2	31.5↑	39.8↑	66.5	4.6		19↑	42.4↑
P6	F	5Y	12.4			11.3↓	53.5↑	35↑		6↓	19.7	35.2↑	39.1↑	40.5↓	6.1↓		9.6↑	23.4↑
P7	F	7Y	3.2			16.6↓	72.9↑	10.4↑		15.1↓	47.1↑	20.8↑	17	31.4↓	13.3		17.7↑	72.9↑
P8	M	5M	4			52.1	36	11↑		20.8↓	30.3	30.7↑	18.3	0.1↓	2.3		1.2	72.6↑
P9	M	5Y	4.7			3.1↓	84.5↑	12.4↑		20.4↓	57.7↑	12.5	9.4	63.4	15		15.5↑	73.7↑
P10	M	8Y	5.8			15.1↓	72.4↑	12.5↑		10.6↓	64.8↑	17.9↑	6.7	29.8↓	7.8		10.6↑	52.7↑
P11	M	10Y	5.2			5.9↓	43.9	50.1↑		7.4↓	31.9	33↑	27.7↑	42.9↓	10.1		22.3↑	1.8
P12	M	5Y	3.9			17.1↓	77.1↑	5.4		14.5↓	60↑	14.7↑	10.8	48.7	6.8↓		6.2	75.4↑
P13	M	6Y	14.5			12.2↓	46.7	35.3↑		5.2↓	23.7	42.7↑	28.4	77.4	4.3↓		9.1	71.3↑
P14	F	5Y	3.1			25.2↓	63↑	11.5↑		9.3↓	9.7	39.4↑	41.7↑	78.7	12		17.1↑	46.5↑
P15	M	6Y	22.2			19.1↓	34	11.5↑		14.3↓	35.5↑	20.8↑	29.4↑	79.2	3.5↓		5.4	52.5↑
P22	F	7Y	2.49	1.47	3.44 ↓	36.91 ↓	49.4	12.93 ↑	0.8	16.26 ↓	14.26	6.48	62.99 ↑	89.41 ↑	1.4 ↓	2.87 ↓	0.8	79.05 ↑
P25	F	17Y	4.75	2.15	2.15↓	26↓	61.14↑	12.72	0.13	40.52	31.65	9.92	17.91	36.5↓	34.26↑	5.66	11.5 ↑	42.2 ↑
P27	F	10Y	3.2	0.94	2.43 ↓	10.51 ↓	74.7 ↑	14.53 ↑	0.26	14.35 ↓	46.96 ↑	18.41 ↑	20.28	83.4 ↑	2.5 ↓	5.46	1.01	90.28 ↑
P30	F	6Y	5.02	0.18 ↓	6.64 ↓	17.23 ↓	61.19 ↑	21.19 ↑	0.39	5.15 ↓	25.37	52.36 ↑	17.12	69.9	14.51	3.99	1.09	38.33 ↑

P31	M	6Y	9.59			19.48↓	16.4	59.88↑	27.99↑	4.23↓	4	52.67↑	51.96↑	79.8	15.94		0.73	47.26↑
P32	M	13Y	13.01	0.51 ↓	14.46	7.3 ↓	39	50.41 ↑	3.25 ↑	4.11 ↓	20.72	52.48 ↑	22.69	26.51 ↓	27.68 ↑	2.14 ↓	8.19 ↑	13.25 ↑
P33	M	4Y	8.66	1.27	5.03 ↓	15.06 ↓	54 ↑	18.63 ↑	12.3 ↑	9.89 ↓	24.36	14.86 ↑	50.88 ↑	88.41 ↑	2.96 ↓	1.17 ↓	2.46	62.4 ↑
P35	M	2Y	7.59	0.23 ↓	10.25	26.45 ↓	61.66 ↑	11.59 ↑	0.3	11.58 ↓	19.68	49.97 ↑	18.77	87.83 ↑	1.33 ↓	1.39 ↓	0.51	53.49 ↑
P36	M	9Y	13.82	0.83	9.56	14.15 ↓	55.89 ↑	27.36 ↑	2.6 ↑	13.47 ↓	31.98	36.74 ↑	17.8	67.1	11.88	14.53	12.51 ↑	39.5 ↑
P37	F	4Y	8.54	0.47 ↓	17.43	33.81 ↓	47.3	18.22 ↑	0.67 ↑	14.49 ↓	29.07	38.6 ↑	17.83	88.23 ↑	1.07 ↓	2.16 ↓	0.74	93.18 ↑
P38	F	5Y	3.21	1	2.01 ↓	8.45 ↓	81.46 ↑	9.89 ↑	0.21	18.45 ↓	63.27 ↑	11.36	6.92	38.16 ↓	30.46 ↑	2.83 ↓	16.97 ↑	50.14 ↑
P39	M	5Y	5.99	1.55	4.2 ↓	22.77 ↓	71.76 ↑	5.4	0.07	29.41 ↓	53.3 ↑	14.64 ↑	2.65	42.41 ↓	32.9 ↑	5.31	32.41 ↑	10.07
P40	F	8Y	5.06	0.72	5.48↓	19.84↓	44	34.8↑	1.4	14.75↓	19.13	24.89↑	41.23↑	58	21.66	7.35	11.35↑	28.05↑
P41	M	11Y	2.23	1.39	1.16↓	13.4↓	73.79↑	12.81↑	0↓	15.53↓	49.69↑	25.43↑	9.35	69.6	3.95↓	2.38↓	2.52	64.92↑
P42	M	8Y	24.53	1.08	14.6	37.75↓	46.7	14.31↑	1.26	39.08↓	31.6	6.42	22.9↑	66.4	10.83	17.32	4.65	65.89↑

Y, years; M, male; F, female; DNT, double negtive T cell; NAÏVE, naive T cells (CD27+CD45RA+); CM, central memory (CD27+CD45RA-); EM, effector memory (CD27-CD45RA-); TEMRA, terminally differentiated effector memory T cells (CD27-CD45RA+); MB, memory B cells(CD19+CD27+); MZB, marginal-zone B cells(CD19+CD27+IgD+); PB, plasmablast(CD19+CD38high+); TRB, transitional B cells(CD19+CD38high+CD24high+).

Table S3 Statistical results of immunological tests in APDS1 patietns with or without autoimmunity

	AD(M(P25,P75))	NAD(M(P25,P75))	P value
NK cells%	24.260%(15.0%,32.1%)	17.040%(11.0%,31.5%)	0.331
NK cell counts	360.070(160.3,640.0)	279.340(190.0,741.8)	0.791
CD3+ T cells%	65.640%(56.1%,77.6%)	64.000%(53.6%,73.5%)	0.587
CD3+ T cell counts	854.000(549.0,1433.9)	1302.000(968.1,1543.9)	0.039*
DNT	5.040%(3.2%,6.7%)	7.590%(3.3%,14.5%)	0.19
CD4+ T cells%	22.850%(16.4%,32.9%)	22.000%(12.8%,26.7%)	0.376
CD4+ T cell counts	327.640(233.5,480.8)	400.200(325.9,617.8)	0.146
CD4 NAÏVE	15.080%(10.2%,17.9%)	19.100% (11.3%,26.4%)	0.275
CD4 CM	58.515% (44.0%,73.1%)	53.500% (46.7%,71.8%)	0.694
CD4 EM	16.580% (12.7%,34.3%)	12.400% (11.0%,35.0%)	0.407
CD4 TEMRA	0.800% (0.2%,2.9%)	0.485% (0.2,7%.9%)	0.724
CD8+ T cells%	37.390%(29.9%,46.0%)	34.070%(23.8%,47.6%)	0.487
CD8+ T cell counts	451.890(290.0,716.1)	669.880(526.5,962.5)	0.053
CD8 NAÏVE	14.425% (9.3%,15.7%)	14.490% (9.3%,20.4%)	0.527
CD8 CM	31.940% (23.4%,47.7%)	29.070% (19.7%,35.5%)	0.275
CD8 EM	22.845% (14.8%,34.7%)	31.500% (14.6%,39.4%)	0.471
CD8 TEMRA	17.855% (12.1%,31.1%)	22.900% (17.8%,39.8%)	0.383
B cells %	6.930%(5.3%,12.0%)	12.000%(6.2%,18.7%)	0.056
B cells counts	91.970(57.3,187.3)	248.330(167.2,502.4)	0.003**
Naïve B	53.360% (31.0%,73.3%)	71.900% (42.4%,79.2%)	0.295
Memory B	10.990% (3.7%,23.2%)	6.100% (3.3%,15.0%)	0.407
Marginal zone B	3.990% (2.3%,6.5%)	2.830% (1.8%,11.3%)	0.739
Plasmablasts	9.395% (2.1%,13.8%)	9.100% (1.2%,17.0%)	0.827
Transitional B	47.450% (24.4%,73.5%)	52.500% (46.5%,71.3%)	0.513
IgG(g/L)	10.700(6.6,14.5)	6.600(2.1,9.2)	0.031*
IgA(g/L)	0.990(0.2,1.2)	0.540(0.1,1.3)	0.677
IgM(g/L)	3.840(2.1,5.6)	3.660(2.1,4.9)	0.686
IgE(g/L)	28.470(16.9,49.3)	28.000(6.6,40.7)	0.237

αβDNT, double negtive T cell(CD3+CD4-CD8-TCRαβ+); γδT(CD3+TCRγδ+); NAÏVE, naive T cells (CD27+CD45RA+); CM, central memory (CD27+CD45RA-); EM, effector memory (CD27-CD45RA-); TEMRA, terminally differentiated effector memory T cells (CD27-CD45RA+); Naive B(CD19+CD27-IgD+); Memory B(CD19+CD27+); Marginal zone B(CD19+CD27+IgD+); Plasmablasts(CD19+CD38high+); Transitional B(CD19+CD38high+CD24high+).* $p<0.05$ ** $p<0.01$

Table S4. Overview of the 6 panels for immunophenotyping of human whole blood

Fluorochrome	FITC	PerCP/Cy5.5	PE	PE/CF594	PE/Cy7	APC	AF700	APC/Fire750	BV421	BV510	BV605
T eff/mem and Treg cells Panel											
Specificity	CCR7	CD3	CD95	CD25	HLADR	CD39	CD45RA	CD4	CD127	CD45	CD8
T cells activation Panel											
Specificity	CD85j	CD3	PD-1	CD69	CD28	CD57	HLADR	CD4	CD38	CD45	CD8
Th,Tc,Tfh and Treg cells Panel											
Specificity	CXCR5	CD3	ICOS	CD25	CXCR3	CCR6	CD127	CD4	CCR4	CD45	CD8
B cells Panel											
Specificity	CD21	CD20	CD24	CD11c	IgD	CD38	IgG	CD19	CD27	CD45	IgM

Table S5. Immune cell subset in APDS1 patients versus controls

	Th1	Th2	Th9	Th17	Th17Th1	Tfh	Tfr	Tregs	Activated Tregs	Memory Tregs	Naive Tregs	CD11c+ B	CD21lo B
C1	70.4 %	68.4 %	2.47 %	31.1 %	29.0 %	6.50 %	8.52 %	5.50 %	30.2 %	38.4 %	28.1 %	13.6 %	4.69 %
C2	90.7 %	53.1 %	2.19 %	46.3 %	8.81 %	9.94 %	8.99 %	5.01 %	32.3 %	40.6 %	19.9 %	13.9 %	3.46 %
C3	51.7 %	53.9 %	18.9 %	44.7 %	48.3 %	2.77 %	1.61 %	7.32 %	27.3 %	29.4 %	40.3 %	19.5 %	9.71 %
C4	82.9 %	78.4 %	3.23 %	20.6 %	16.8 %	7.03 %	2.86 %	7.98 %	26.9 %	39.1 %	32.4 %	10.1 %	3.93 %
C5	59.3 %	64.0 %	3.71 %	35.2 %	39.7 %	5.38 %	8.81 %	5.23 %	26.3 %	39.6 %	28.3 %	16.8 %	4.61 %
C6	56.6 %	63.2 %	12.2 %	36.2 %	42.3 %	4.68 %	5.10 %	4.93 %	22.6 %	64.3 %	9.89 %	7.63 %	7.40 %
C7	68.8 %	55.6 %	7.27 %	43.3 %	30.2 %	4.09 %	5.26 %	3.98 %	70.9 %	19.8 %	8.72 %	10.5 %	3.90 %
C8	86.3 %	66.5 %	1.94 %	32.4 %	13.4 %	6.38 %	4.46 %	8.97 %	58.1 %	18.1 %	19.3 %	13.4 %	11.2 %
C9	66.9 %	44.3 %	3.41 %	54.7 %	32.1 %	6.85 %	5.95 %	4.22 %	41.9 %	29.6 %	22.3 %	8.63 %	11.5 %
P36	57.1 %	71.9 %	11.3 %	27.6 %	42.6 %	20.2 %	15.8 %	5.92 %	37.4 %	41.7 %	17.5 %	14.5 %	45.2 %
P1	73.8 %	54.7 %	7.43 %	44.4 %	25.9 %	19.4 %	14.0 %	3.30 %	27.0 %	62.4 %	9.55 %	49.2 %	18.8 %
P27	43.6 %	41.8 %	23.3 %	56.8 %	55.1 %	38.8 %	17.0 %	6.62 %	46.1 %	47.8 %	2.61 %	13.3 %	7.14 %
P25	69.9 %	70.6 %	11.9 %	26.3 %	28.3 %	26.7 %	16.6 %	5.00 %	33.3 %	54.3 %	4.94 %	48.8 %	13.3 %
P7	82.7 %	61.5 %	7.62 %	36.2 %	16.4 %	23.3 %	12.1 %	4.85 %	63.7 %	29.8 %	1.75 %	26.4 %	10.3 %
P22	65.8 %	48.3 %	8.90 %	50.4 %	33.2 %	13.6 %	12.3 %	3.12 %	22.0 %	69.5 %	5.08 %	15.6 %	8.86 %
P17	81.4 %	61.5 %	4.89 %	37.6 %	18.3 %	16.5 %	20.0 %	4.82 %	33.1 %	58.8 %	6.05 %	38.4 %	11.4 %
P39	67.3 %	60.6 %	6.71 %	38.2 %	32.2 %	33.4 %	11.3 %	3.82 %	25.9 %	46.8 %	26.5 %	26.6 %	19.6 %

Table S6. MFI of different makers in T cell subsets

	HC1	HC2	HC3	HC4	HC5	HC6	HC7	HC8	HC9	P39	P36	P1	P27	P25	P7	P22	P17
CD95 in DNT cells	49881	50799	42402	51110	14503	38685	31947	28767	48099	53342	61666	49881	81876	222433	90126	54332	58140
CD85j in DNT cells	2353	4164	1763	2706	2314	2170	1932	2785	3862	2027	3940	1781	2946	3243	2189	3873	2333
PD-1 in DNT cells	83.7	356	3730	797	-41.9	3793	1628	503	52.3	545	178	1628	1	8085	2400	45800	199
CD69 in DNT cells	3013	1960	11570	9776	9069	8395	14730	7939	8240	2849	6673	11443	6781	5233	7875	4207	6495
CD28 in DNT cells	3851	298	3527	6697	2343	7726	932	1363	714	832	569	13542	3201	3233	1585	5802	1866
CD57 in DNT cells	77.2	83	67.6	-9.65	154	116	203	193	222	2309	512	19.3	2350	396	34879	75334	803
HLADR in DNT cells	787	3048	714	397	469	978	1115	1060	841	3027	3058	1051	1941	542	4838	361	2725
CD38 in DNT cells	4256	9391	6363	13153	1446	632	6498	1716	2528	13411	10770	2936	20681	8088	27894	8968	6651
CD95 in CD4+ T cells	11808	75622	22706	23201	6027	109423	89262	6682	14325	74200	102542	69884	108714	113416	124701	74435	76345
CD85j in CD4+ T cells	1133	2179	1317	2151	1317	1847	1188	1474	823	1372	996	1904	1400	2122	1979	2518	1604
PD-1 in CD4+ T cells	1030	4064	3908	1900	209	4644	5749	818	945	13392	12982	23345	23642	26017	48452	42713	24249
CD69 in CD4+ T cells	1313	1559	2551	3143	2081	4155	3593	2147	2990	271	2026	3718	2849	3730	5445	2381	4024
CD28 in CD4+ T cells	14325	16347	13249	13575	15861	17964	23139	17198	15315	20195	17964	11262	26312	12870	18673	13741	10770
CD57 in CD4+ T cells	-29	124	381	1	116	125	222	1	174	900	19.3	174	77.2	8240	2517	45621	328
HLADR in CD4+ T cells	-117	316	-135	-198	-442	189	352	-271	-108	641	-27.1	171	696	1	678	996	723
CD38 in CD4+ T	3096	7278	3422	3140	2062	1033	6513	1972	1931	5358	5679	4063	12179	9241	11808	1072	5789

cells	1		8	5	6			4	5							0	
CD95 in CD8+ T cells	20195	51110	47092	49129	15508	22104	42782	32038	24497	40917	54835	49279	55003	78807	59597	49279	44879
CD85j in CD8+ T cells	2074	4680	4974	3601	2489	2916	1941	7080	2275	1866	1894	2986	1819	2815	3058	4383	2735
PD-1 in CD8+ T cells	4478	3034	3883	8830	3717	5133	10813	1221	2502	17390	5622	10129	5960	11840	5104	4547	3883
CD69 in CD8+ T cells	8598	4422	23255	16400	16545	16496	21644	7854	17294	9069	15470	22750	13755	15880	13447	16209	17090
CD28 in CD8+ T cells	5125	524	705	1539	8144	3917	1179	687	3442	2275	1326	1941	3884	2626	1941	768	923
CD57 in CD8+ T cells	164	709	993	319	183	270	803	386	338	473	232	852	299	367	35993	22956	3240
HLADR in CD8+ T cells	235	1492	732	-54.1	144	406	1170	696	180	1632	1465	868	1613	1206	3078	578	2401
CD38 in CD8+ T cells	7430	12534	27052	29422	2247	4900	13411	6113	5652	6605	5293	4631	13910	6099	22402	12296	5844

Table S7. The result of Dunn test

Antigen	(I)Group	(J)Group	(I)Median	(J)Meidan	Value(I-J)	p
Histone H2B	Control	Patient	148.105	306.484	-158.379	0.010*
	Control	Patient&AD	148.105	522.377	-374.272	0.001***
	Patient	Patient&AD	306.484	522.377	-215.894	0.182
Histone H3	Control	Patient	87.116	147.143	-60.027	0.164
	Control	Patient&AD	87.116	222.755	-135.639	0.008**
	Patient	Patient&AD	147.143	222.755	-75.612	0.135
Jo-1	Control	Patient	1779.164	2298.507	-519.342	0.162
	Control	Patient&AD	1779.164	3548.445	-1769.28	0.001**
	Patient	Patient&AD	2298.507	3548.445	-1249.94	0.045*
KU (P70/P80)	Control	Patient	665.413	1058.934	-393.521	0.268
	Control	Patient&AD	665.413	2848.265	-2182.85	0.004**
	Patient	Patient&AD	1058.934	2848.265	-1789.33	0.051
La/SSB	Control	Patient	688.736	1065.962	-377.225	0.43
	Control	Patient&AD	688.736	2248.159	-1559.42	0.008**
	Patient	Patient&AD	1065.962	2248.159	-1182.2	0.048*
MDA5	Control	Patient	772.076	2117.153	-1345.08	0.064
	Control	Patient&AD	772.076	3614.732	-2842.66	0.004**
	Patient	Patient&AD	2117.153	3614.732	-1497.58	0.175
Mi-2	Control	Patient	176.359	288.963	-112.604	0.259
	Control	Patient&AD	176.359	393.433	-217.074	0.014*
	Patient	Patient&AD	288.963	393.433	-104.47	0.135
PCNA	Control	Patient	871.369	1157.094	-285.724	0.154
	Control	Patient&AD	871.369	1946.427	-1075.06	0.002**
	Patient	Patient&AD	1157.094	1946.427	-789.333	0.053
PL-12	Control	Patient	714.599	635.549	79.05	0.891
	Control	Patient&AD	714.599	2280.771	-1566.17	0.010*
	Patient	Patient&AD	635.549	2280.771	-1645.22	0.009**
PL-7	Control	Patient	602.271	808.482	-206.21	0.354
	Control	Patient&AD	602.271	1135.722	-533.451	0.008**
	Patient	Patient&AD	808.482	1135.722	-327.24	0.062
PM/Scl 100	Control	Patient	321.787	414.175	-92.388	0.292
	Control	Patient&AD	321.787	877.106	-555.318	0.004**
	Patient	Patient&AD	414.175	877.106	-462.931	0.048*
Ribo Phosphoprotein P0	Control	Patient	152.242	194.668	-42.426	0.324
	Control	Patient&AD	152.242	281.506	-129.263	0.010*
	Patient	Patient&AD	194.668	281.506	-86.838	0.084
Ribo Phosphoprotein P2	Control	Patient	243.886	651.802	-407.916	0.076
	Control	Patient&AD	243.886	1890.672	-1646.79	0.001***
	Patient	Patient&AD	651.802	1890.672	-1238.87	0.066
Ro/SSA (52 Kda)	Control	Patient	681.727	1798.631	-1116.9	0.019*
	Control	Patient&AD	681.727	3725.084	-3043.36	0.007**
	Patient	Patient&AD	1798.631	3725.084	-1926.45	0.421
Ro/SSA (60 Kda)	Control	Patient	580.962	1364.173	-783.211	0.005**
	Control	Patient&AD	580.962	4634.626	-4053.66	0.000***
	Patient	Patient&AD	1364.173	4634.626	-3270.45	0.129
Scl-70/Topoisomerase I	Control	Patient	318.361	236.059	82.302	0.806
	Control	Patient&AD	318.361	507.509	-189.148	0.010**
	Patient	Patient&AD	236.059	507.509	-271.449	0.020*
Sm/RNP	Control	Patient	95.391	147.669	-52.278	0.139
	Control	Patient&AD	95.391	256.195	-160.805	0.012*

	Patient	Patient&AD	147.669	256.195	-108.526	0.198
	Control	Patient	195.24	236.594	-41.354	0.237
SmD	Control	Patient&AD	195.24	334.376	-139.136	0.014*
	Patient	Patient&AD	236.594	334.376	-97.782	0.141
	Control	Patient	419.082	510.664	-91.583	0.837
TIF1 gama	Control	Patient&AD	419.082	1070.339	-651.257	0.025*
	Patient	Patient&AD	510.664	1070.339	-559.674	0.019*
	Control	Patient	574.237	764.509	-190.271	0.369
U1-snRNP 68/70	Control	Patient&AD	574.237	2341.397	-1767.16	0.007**
	Patient	Patient&AD	764.509	2341.397	-1576.89	0.053
	Control	Patient	520.704	622.303	-101.599	0.237
U1-snRNP A	Control	Patient&AD	520.704	1265.477	-744.773	0.014*
	Patient	Patient&AD	622.303	1265.477	-643.174	0.141
	Control	Patient	184.366	233.215	-48.848	0.12
U1-snRNP B/B’	Control	Patient&AD	184.366	709.415	-525.048	0.007**
	Patient	Patient&AD	233.215	709.415	-476.2	0.154
	Control	Patient	259.424	359.572	-100.148	0.155
U1-snRNP C	Control	Patient&AD	259.424	433.929	-174.505	0.009**
	Patient	Patient&AD	359.572	433.929	-74.357	0.154
	Control	Patient	1064.451	1533.025	-468.574	0.092
B-actin	Control	Patient&AD	1064.451	3783.819	-2719.37	0.001***
	Patient	Patient&AD	1533.025	3783.819	-2250.8	0.041*
	Control	Patient	749.128	1095.445	-346.317	0.066
BPI	Control	Patient&AD	749.128	1610.359	-861.231	0.002**
	Patient	Patient&AD	1095.445	1610.359	-514.913	0.112
	Control	Patient	788.596	777.439	11.157	0.245
LC1	Control	Patient&AD	788.596	1632.806	-844.21	0.003**
	Patient	Patient&AD	777.439	1632.806	-855.367	0.053
	Control	Patient	856.059	1316.868	-460.809	0.062
M2	Control	Patient&AD	856.059	2011.879	-1155.82	0.015*
	Patient	Patient&AD	1316.868	2011.879	-695.011	0.37
	Control	Patient	329.414	501.73	-172.316	0.073
MPO	Control	Patient&AD	329.414	1321.717	-992.303	0.000***
	Patient	Patient&AD	501.73	1321.717	-819.987	0.034*
	Control	Patient	883.82	1060.645	-176.825	0.339
Myosin	Control	Patient&AD	883.82	8278.278	-7394.46	0.009**
	Patient	Patient&AD	1060.645	8278.278	-7217.63	0.073
	Control	Patient	396.031	671.198	-275.167	0.285
Neuropilin-1	Control	Patient&AD	396.031	983.177	-587.146	0.011*
	Patient	Patient&AD	671.198	983.177	-311.979	0.102
	Control	Patient	1627.7	4367.043	-2739.34	0.064
TTG	Control	Patient&AD	1627.7	17471.375	-15843.7	0.000***
	Patient	Patient&AD	4367.043	17471.375	-13104.3	0.048*
	Control	Patient	1308.347	1862.089	-553.742	0.249
Collagen VI	Control	Patient&AD	1308.347	3629.372	-2321.02	0.012*
	Patient	Patient&AD	1862.089	3629.372	-1767.28	0.123
	Control	Patient	660.867	941.347	-280.48	0.077
GM-CSF	Control	Patient&AD	660.867	2735.682	-2074.82	0.005**
	Patient	Patient&AD	941.347	2735.682	-1794.33	0.182
	Control	Patient	543.224	626.44	-83.217	0.086
IL-15	Control	Patient&AD	543.224	1136.545	-593.321	0.005**
	Patient	Patient&AD	626.44	1136.545	-510.104	0.175

Cardolipin	Control	Patient	10725.146	13679.14	-2953.99	0.046*
	Control	Patient&AD	10725.146	21756.479	-11031.3	0.006**
	Patient	Patient&AD	13679.14	21756.479	-8077.34	0.26
Phosphatidyl-serine (PS)	Control	Patient	509.448	1055.645	-546.197	0.025*
	Control	Patient&AD	509.448	2496.942	-1987.49	0.010*
	Patient	Patient&AD	1055.645	2496.942	-1441.3	0.462
Laminin	Control	Patient	245.549	348.558	-103.009	0.187
	Control	Patient&AD	245.549	495.799	-250.25	0.014*
	Patient	Patient&AD	348.558	495.799	-147.241	0.175
AQP4	Control	Patient	3569.746	5393.261	-1823.52	0.133
	Control	Patient&AD	3569.746	12808.144	-9238.4	0.001***
	Patient	Patient&AD	5393.261	12808.144	-7414.88	0.038*
S100	Control	Patient	2143.648	3456.482	-1312.83	0.052
	Control	Patient&AD	2143.648	11396.447	-9252.8	0.002**
	Patient	Patient&AD	3456.482	11396.447	-7939.97	0.123
IA2	Control	Patient	325.066	409.1	-84.034	0.213
	Control	Patient&AD	325.066	631.642	-306.575	0.004**
	Patient	Patient&AD	409.1	631.642	-222.542	0.066
Calprotectin	Control	Patient	1685.583	2117.207	-431.624	0.761
	Control	Patient&AD	1685.583	5589.178	-3903.6	0.013*
	Patient	Patient&AD	2117.207	5589.178	-3471.97	0.029*
CRP	Control	Patient	4656.195	5175.499	-519.305	0.336
	Control	Patient&AD	4656.195	8029.801	-3373.61	0.002**
	Patient	Patient&AD	5175.499	8029.801	-2854.3	0.021*
EBNA1	Control	Patient	11420.56	46546.105	-35125.5	0.005**
	Control	Patient&AD	11420.56	45373.815	-33953.3	0.045*
	Patient	Patient&AD	46546.105	45373.815	1172.29	0.818
Factor B	Control	Patient	837.835	1668.561	-830.726	0.035*
	Control	Patient&AD	837.835	2075.278	-1237.44	0.014*
	Patient	Patient&AD	1668.561	2075.278	-406.717	0.462
Hemocyanin	Control	Patient	2496.912	3074.615	-577.703	0.088
	Control	Patient&AD	2496.912	5687.483	-3190.57	0.003**
	Patient	Patient&AD	3074.615	5687.483	-2612.87	0.112
Insulin	Control	Patient	27.977	75.724	-47.747	0.156
	Control	Patient&AD	27.977	193.201	-165.224	0.006**
	Patient	Patient&AD	75.724	193.201	-117.477	0.123
LPS	Control	Patient	3668.562	10111.273	-6442.71	0.096
	Control	Patient&AD	3668.562	16123.069	-12454.5	0.006**
	Patient	Patient&AD	10111.273	16123.069	-6011.8	0.168

* p<0.05 ** p<0.01 *** p<0.001

Table S8. Immunological tests of P39

	CD16CD56%	CD16CD56 counts cells/ul	CD3%	CD3counts cells/ul	CD19%	CD19counts cells/ul	CD4%	CD4counts cells/ul	CD8%	CD8counts cells/ul
Before treatment	31.55	743.31	18.00	527.50	2.72	64.04	39	918.79	22.35	526.47
After treatment	23.08	461.55	58.04	1160	16.94	338.75	34.27	685.24	21.39	427.63

Table S9 Basic information of patients and controls

ID	Age	Sex
P1	23Y	M
P7	15Y	F
P13	11Y	M
P17	12Y	M
P22	7Y	F
P25	11Y	F
P27	10Y	F
P30	6Y	F
P36	10Y	M
P37	4Y	F
P38	6Y	F
P39	5Y	M
P40	8Y	F
P41	11Y	M
P42	8Y	M
HC1	24Y	M
HC2	14Y	F
HC3	7Y	F
HC4	11Y	F
HC5	10Y	F
HC6	10Y	M
HC7	5Y	M
HC8	6Y	M
HC9	11Y	F
C1	14Y	M
C2	4Y	F
C3	10Y	M
C4	14Y	F

C5	8Y	M
C6	8Y	M
C7	10Y	M
C8	2Y	F
C9	6Y	M
C10	14Y	M
C11	11Y	F
C12	12Y	F

Fig.S1 Comparison of serum IgM levels between AD and NAD group.

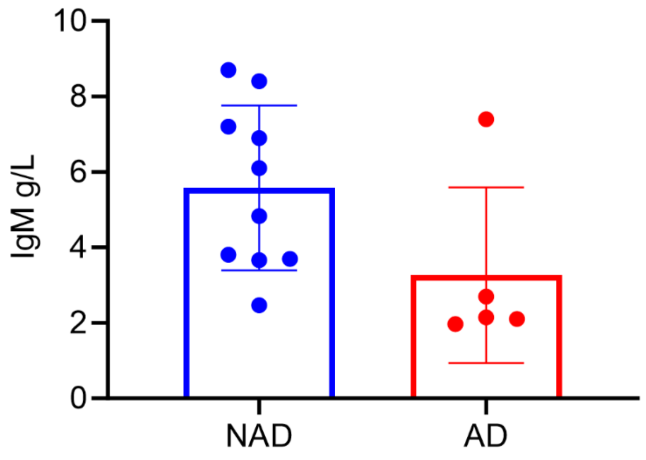

Supplement: Supplementary file 1 — Supplementary Material 1 [file 10875_2024_1705_MOESM1_ESM.pdf]
