# Supplementary material for: Phenotypic and Immunological Characterization of Patients with Activated PI3Kδ Syndrome 1 Presenting with Autoimmunity
Source: J Clin Immunol. 2024 Apr 18;44(4):102. doi: 10.1007/s10875-024-01705-w (PMC11026262; doi:10.1007/s10875-024-01705-w)
Supplement: Supplementary file 2 — Supplementary Material 2 [file 10875_2024_1705_MOESM2_ESM.docx]

**Fig.S1 Comparison of serum IgM levels between AD and NAD group.**
