# Supplementary figures and images for: Phenotypic and Immunological Characterization of Patients with Activated PI3Kδ Syndrome 1 Presenting with Autoimmunity
Source: J Clin Immunol. 2024 Apr 18;44(4):102. doi: 10.1007/s10875-024-01705-w (PMC11026262; doi:10.1007/s10875-024-01705-w)

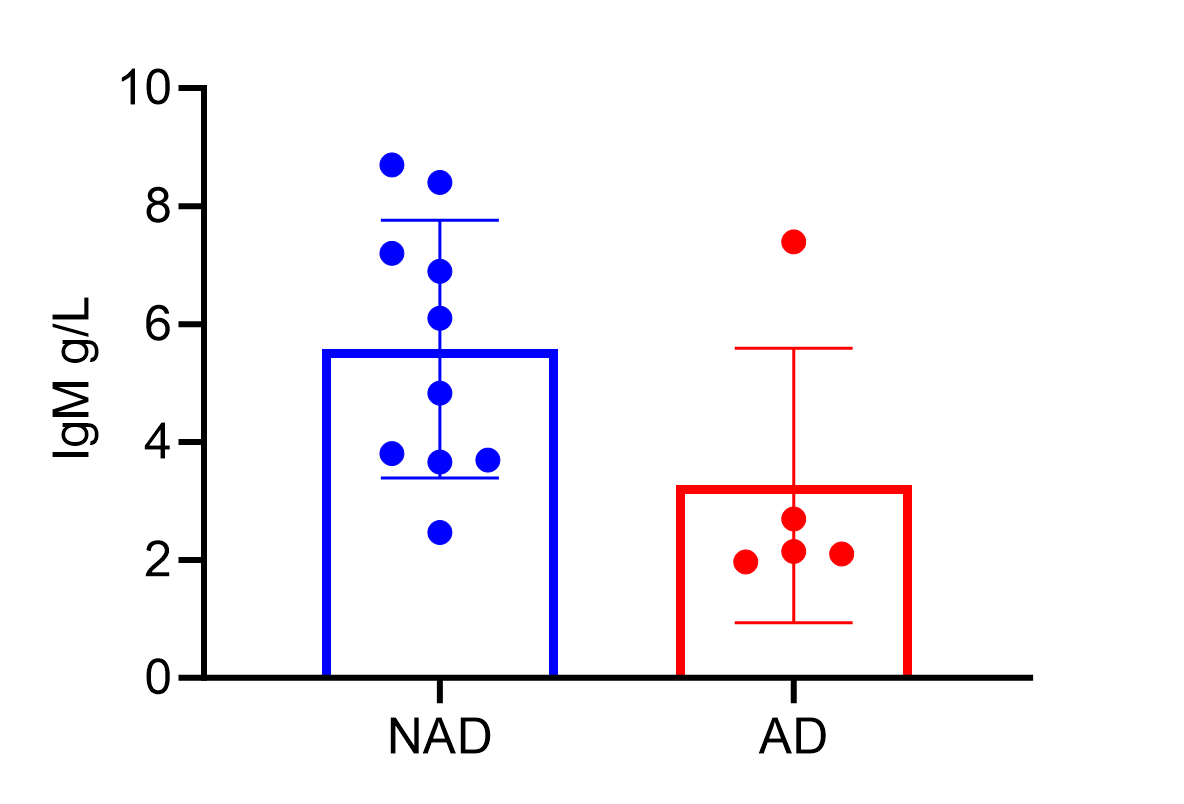

Supplement: Supplementary file 3 — Supplementary Material 3 [file 10875_2024_1705_MOESM3_ESM.tif]
